# Nitrogen Level Impacts the Dynamic Changes in Nitrogen Metabolism, and Carbohydrate and Anthocyanin Biosynthesis Improves the Kernel Nutritional Quality of Purple Waxy Maize

**DOI:** 10.3390/plants13202882

**Published:** 2024-10-15

**Authors:** Wanjun Feng, Weiwei Xue, Zequn Zhao, Haoxue Wang, Zhaokang Shi, Weijie Wang, Baoguo Chen, Peng Qiu, Jianfu Xue, Min Sun

**Affiliations:** 1 Sorghum Research Institute, Shanxi Agricultural University, Jinzhong 030600, China; fengwj123@sxau.edu.cn (W.F.); wangweijie0628@163.com (W.W.); qiupeng072954@163.com (P.Q.); 2 College of Agriculture, Shanxi Agricultural University, Jinzhong 030801, China; z20223184@stu.sxau.edu.cn (W.X.); zhaozequnsd@163.com (Z.Z.); z20223217@stu.sxau.edu.cn (H.W.); shishizhaokang@163.com (Z.S.); bgchen108@sina.com (B.C.)

**Keywords:** nitrogen application, anthocyanin accumulation, enzyme activity, kernel quality, purple waxy corn

## Abstract

Waxy corn is a special type of maize primarily consumed as a fresh vegetable by humans. Nitrogen (N) plays an essential role in regulating the growth progression, maturation, yield, and quality of waxy maize. A reasonable N application rate is vital for boosting the accumulation of both N and carbon (C) in the grains, thereby synergistically enhancing the grain quality. However, the impact of varying N levels on the dynamic changes in N metabolism, carbohydrate formation, and anthocyanin synthesis in purple waxy corn kernels, as well as the regulatory relationships among these processes, remains unclear. To explore the effects of varying N application rates on the N metabolism, carbohydrate formation, and anthocyanin synthesis in kernels during grain filling, a two-year field experiment was carried out using the purple waxy maize variety Jinnuo20 (JN20). This study examined the different N levels, specifically 0 (N0), 120 (N1), 240 (N2), and 360 (N3) kg N ha^−1^. The results of the analysis revealed that, for nearly all traits measured, the N application rate of N2 was the most suitable. Compared to the N0 treatment, the accumulation and content of anthocyanins, total nitrogen, soluble sugars, amylopectin, and C/N ratio in grains increased by an average of 35.62%, 11.49%, 12.84%, 23.74%, 13.00%, and 1.87% under N2 treatment over five filling stages within two years, respectively, while the harmful compound nitrite content only increased by an average of 30.2%. Correspondingly, the activities of related enzymes also significantly increased and were maintained under N2 treatment compared to N0 treatment. Regression and correlation analysis results revealed that the amount of anthocyanin accumulation was highly positively correlated with the activities of phenylalanine ammonia-lyase (PAL) and flavanone 3-hydroxylase (F3H), but negatively correlated with anthocyanidin synthase (ANS) and UDP-glycose: flavonoid-3-O-glycosyltransferase (UFGT) activity, nitrate reductase (NR), and glutamine synthetase (GS) showed significant positive correlations with the total nitrogen content and lysine content, and a significant negative correlation with nitrite, while soluble sugars were negatively with ADP-glucose pyrophosphorylase (AGPase) activity, and amylopectin content was positively correlated with the activities of soluble starch synthase (SSS), starch branching enzyme (SBE), and starch debranching enzyme (SDBE), respectively. Furthermore, there were positive or negative correlations among the detected traits. Hence, a reasonable N application rate improves purple waxy corn kernel nutritional quality by regulating N metabolism, as well as carbohydrate and anthocyanin biosynthesis.

## 1. Introduction

Waxy maize (*Zea mays* L. var. *ceratina* Kulesh), commonly referred to as sticky corn, is a maize subspecies that originated in southwestern China in the early 20th century [1]. Due to a mutation in the *waxy* gene (*Wx*), nearly all starch synthesized in waxy corn grains consists of amylopectin [2]. It has a distinctive flavor, high nutritional content, economic value, and ease of processing; thus, it has become favored by consumers and farmers in recent years, particularly in Asia [3,4]. Purple waxy corn, characterized by its striking purple kernels and unique amylopectin-rich texture, is a specialized maize variety with both nutritional and commercial importance [5]. Anthocyanins are the pigments that impart the kernels with their bright purple coloration, not only contributing to the visual appeal but also offering valuable antioxidant properties that can benefit human health, such as inhibiting tumors, regulating lipid and carbohydrate metabolism, and yielding anti-fatigue and anti-aging properties [6,7]. Anthocyanins are water-soluble natural pigments belonging to the flavonoid group, and are commonly found in angiosperms. They are key secondary metabolites that contribute to the red, purple, or blue colors seen in various tissues and organs of higher plants [8]. Thus, the anthocyanin content is an important visible trait with significant applications in agricultural production [9]. Until now, the synthesis pathway of anthocyanin has been relatively well understood, involving numerous enzymes, such as the phenylalanine ammonia-lyase (PAL), chalcone synthase (CHS), chalcone isomerase (CHI), flavanone 3-hydroxylase (F3H), dihydroflavonol4-reductase (DFR), leucoanthocyanidin dioxygenase (LDOX), anthocyanidin synthase (ANS), and UDP-glycose: flavonoid-3-O-glycosyltransferase (UFGT) [10,11], in which DFR is a crucial enzyme for anthocyanin biosynthesis, and is regulated by numerous transcription factors [9]. However, the regulation of anthocyanin biosynthesis is quite intricate, as its content can be influenced by various abiotic stresses, including fungal infections, heavy metal exposure, pH levels, temperature fluctuations, and excessive light [12,13].

The synthesis and storage of storage amino acids, protein, and starch in maize kernels are intricately linked to nitrogen (N) and carbohydrate metabolism. Waxy maize, in particular, stands out for its unique carbohydrate metabolism, lacking amylose production and predominantly consisting of amylopectin. Studies have shown that there is a significant interplay between N and carbohydrate metabolism in maize grains [14,15,16]. Hence, it is believed that there are likely significant differences in carbon and N metabolism in kernels between waxy maize and common maize. Nitrate reductase (NR), nitrite reductase (NiR), glutamine synthetase (GS), and glutamine-2-oxoglutarate aminotransferase (GOGAT or glutamate synthase) play crucial roles in the N metabolism process [17]. Soluble sugars and amylopectins are essential constituents of carbohydrates that significantly influence the composition and functional properties of waxy maize kernels. Soluble starch synthase (SSS), starch branching enzyme (SBE), and starch debranching enzyme (SDBE) are critical for amylopectin biosynthesis in cereal grains, playing pivotal roles in the metabolic pathways governing grain growth and yield production. Moreover, ADP-glucose pyrophosphorylase (AGPase) catalyzes the conversion of ADP-glucose into starch polymers, supplying both the energy and structural components necessary for grain growth [18].

N is an essential nutrient for plant growth and development, serving as a key component in various metabolic pathways that influence crop yield and nutritional quality [19]. In maize, N availability is particularly critical during the kernel filling stages, where it impacts processes such as N metabolism, carbohydrate synthesis, and pigment production [20,21]. N fertilization clearly impacts nitrogen metabolism in plants, affecting protein and amino acid synthesis, along with the translocation and distribution of nitrogen within maize grains [22,23]. Lysine, an indispensable amino acid, is essential for protein quality and feed nutrition [24], which can be also influenced by N availability and contributes to the overall nutritional quality of maize [25]. Moreover, nitrogen availability can affect photosynthetic activity and carbohydrate metabolism in maize grains, which play a vital role in starch and carbohydrate accumulation, ultimately contributing to kernel development and yield production [22]. In addition, N also affects the anthocyanin biosynthesis by regulating the transcript levels of related genes, such as *PAL*, *CHS*, *F3H*, *DFR*, *LDOX*, and *UFGT* [26]. A recent study demonstrated that applying N fertilizer can enhance anthocyanin accumulation in the aboveground parts of purple rice during vegetative growth, although it does not significantly affect the anthocyanin content within the kernel [27]. To date, anthocyanin content profiles in purple waxy maize grains across different harvest periods have been documented [5], but few studies have explored the effects of nitrogen application on anthocyanin accumulation, specifically within purple waxy maize grains.

In our recent study, we treated the year as a factor to capture potential variations arising from environmental and agronomic conditions that can differ across the years. This decision was made to gain a deeper understanding of the effects of nitrogen application on various metabolites and their related enzymes, which are influenced by both genetic and environmental factors [28]. In contrast, in the present study, a unique purple waxy maize commonly consumed by humans was chosen as the experimental material, and we mainly focused on the dynamic variations of anthocyanin, lysine, nitrite, soluble sugars, amylopectin, and their related enzyme activities in the kernels during different filling stages and under different N application doses. Particularly, nitrite, a special intermediate product of N metabolism converted from nitrate by NR, directly influences the safety and nutritional quality of crop products intended for human consumption, as nitrite can be harmful to human health when present in excessive amounts [29]; however, a few studies have found it imperative to monitor the nitrite levels in maize products to date. We hypothesize that variations in nitrogen application rates will result in significant alterations in the concentrations of these metabolites and their related enzymes, specifically affecting nitrite levels. Therefore, this study aims to provide critical insights into the effects of nitrogen management practices on the quality of purple waxy maize, thereby enhancing both its nutritional value and safety for human consumption.

## 2. Materials and Methods

### 2.1. Material Planting and Preparing

The primary purple waxy maize variety, Jinnuo20 (JN20), which is widely cultivated in Shanxi, was selected as the study material. The seeds were procured from Shanxi Dafeng Seed Industry Co., Ltd. (Taiyuan, China). Field experiments were conducted in Dongshan Bottom Village (37°22′28″ N, 12°35′8″ E), Taigu County, Shanxi Province, China, during the 2018 and 2019 growing seasons. The experimental site is situated at an elevation of 791 m above sea level, and the experimental site had a total precipitation and average temperature of 383.0 mm and 21.6 °C in 2018, and 291.6 mm and 25.1 °C in 2019 during the maize growing season, from May to September, respectively. Prior to the commencement of the trial, the field had been afforested for five years without any crop cultivation or fertilizer application. The topsoil (0–20 cm) in the experimental field was characterized as sandy loam with a pH of 6.1. Soil nutrient content before sowing was measured as follows: total N, alkali hydrolysable N, available phosphorus (P), exchangeable potassium (K), and organic matter were 0.66 g kg^−1^, 30.24 mg kg^−1^, 20.02 mg kg^−1^, 114.11 mg kg^−1^ and 18.10 g kg^−1^, respectively.

Maize cultivation commenced in late May in both 2018 and 2019, utilizing a completely randomized design (CRD) with three replicates. Each plot measured 40 m² (4 m × 10 m) and consisted of 8 rows, with a planting density of 60,000 plants per hectare. Four nitrogen (N) application rates were established: 0, 120, 240, and 360 kg ha^−1^ of pure N, designated as N0, N1, N2, and N3, respectively. The fertilizers applied consisted of urea (46% N) for nitrogen, superphosphate (12% P_2_O_5_) for phosphorus, and potassium chloride (60% K_2_O) for potassium. Nitrogen was distributed in a 3:5:2 ratio across the jointing, booting, and anthesis-silking stages. Additionally, there was also a 1:1 ratio of pure potassium (K_2_O) (240 kg ha^−1^) to phosphorus (P_2_O_5_) (120 kg ha^−1^) applied during the sowing and jointing stages. Conventional field management procedures were followed throughout corn cultivation. To prevent cross-pollination, the harvested ears were manually bagged. Sampling was undertaken from 15 to 35 days after pollination (DAP), at five-day intervals. Five evenly developing ears per treatment group were chosen for seed extraction from each of the three replicates. Some seeds were blanched in an oven at 120 °C for 30 min, followed by drying at 80 °C until a constant weight was achieved, and used to determine the biomass, anthocyanin, total N, lysine, nitrite, soluble sugar, and amylopectin content, and the other seeds frozen in liquid N and subsequently stored in an ultra-low temperature refrigerator of −80 °C for detecting the enzyme activities related to anthocyanin biosynthesis, and N and carbohydrate metabolism.

### 2.2. Anthocyanin Quantification

The pH differential method was used to quantify the anthocyanin content in the purple waxy maize seeds [30]. Briefly, 0.2 g of dry grain flour was mixed with 5 mL of a hydrochloric acid and methanol solution (5:95, *v*/*v*), and soaked in water at 50 °C for 1 h, then centrifuged under 10,000× *g* for 10 min. The supernatant was diluted with KCl-HCl buffer with pH 1.0 and citric acid–sodium dihydrogen phosphate buffer with pH 4.5. Finally, a UV spectrophotometer (Shanghai Metash Instruments Co., Ltd., Shanghai, China) was used to measure the optical density (OD) at wavelengths of 510 and 700 nm. The anthocyanin content was calculated as follows:

Grain anthocyanin content (mg/g) = Δ OD × V × N × M/(X × m)

where Δ OD is equal to (OD510 nm–OD700 nm) in pH 1.0-(OD510 nm–OD700 nm) in pH 4.5, V represents the volume of the extraction solution, N denotes the dilution ratio, M indicates the relative molecular weight of cyanidin-3-glucoside, which is 449, X signifies the extinction coefficient of cyanidin-3-glucoside, valued at 29,600, and M refers to the sample mass. The accumulation of anthocyanins in an individual seed was determined by multiplying the anthocyanin content by the dry weight (DW) of that seed.

### 2.3. N Metabolism Product Content Quantification

#### 2.3.1. The Total N Content

The total N content in the seeds was determined using a modified Kjeldahl digestion method [31]. Specifically, 0.5 g of dry grain flour was initially boiled with H_2_SO_4_; for 10 min. Once the solution reaches a uniform brownish-black color, boiling is paused, and the mixture is allowed to cool for 5 min. The heating was then resumed to continue the digestion process. To expedite the oxidation of organic matter, small amounts of hydrogen peroxide (H_2_O_2_) were gradually added in stages until the solution lost its brown hue and became completely clear and transparent. This was followed by an additional heating period of 5–10 min to remove any remaining H_2_O_2_. The resulting solution was then diluted to a final volume of 100 mL with distilled water. Subsequently, 1.0 mL of this diluted solution was mixed with 1 mL of EDTA–methyl red solution, which was adjusted to a pH of 6.0 using 0.3 mol L⁻¹ NaOH. To this mixture, 5 mL of phenol solution and 5 mL of sodium hypochlorite solution were added sequentially. After one hour, the spectra of the mixture at 625 nm was measured using a UV spectrophotometer (Shanghai Metash Instruments Co., Ltd., Shanghai, China). A blank solution was used to calibrate the spectrophotometer to zero. The N content of the samples was then calculated by plotting a working curve based on the measured absorption values.

#### 2.3.2. Nitrite Content

The nitrite content was quantified according a previous method [32]. Briefly, approximately 0.5 g of the sample was placed in a 25 mL beaker and extracted with 3 mL portions of a 0.5% sodium carbonate solution. The extract was then filtered through Whatman No. 41 filter paper, and the filtrate was diluted to a total volume of 25 mL. Specific volumes (1–2 mL) of this diluted solution were transferred to 10 mL calibrated flasks for analysis using standard procedures, which initially yielded negative results. To these solutions, known amounts of nitrite were added and analyzed. Various volumes of a stock solution containing nitrite concentrations ranging from 0.2 to 8.0 µg mL⁻¹ were then dispensed into a series of 10 mL calibrated flasks. Each flask received 1 mL of 0.5% sulfanilic acid and 1 mL of 2 mol L⁻¹ hydrochloric acid, followed by thorough mixing to ensure complete diazotization. Subsequently, 1 mL of 0.5% methyl anthranilate and 2 mL of 2 mol L⁻¹ sodium hydroxide were added to form an azo dye. The solutions were then diluted to 10 mL with water. The absorbance of the resulting red dye was measured at 493 nm against a reagent blank, and a calibration curve was constructed from these measurements.

#### 2.3.3. Lysine Content

The quantification of lysine content followed a previously established method [33]. At the first stage, 1 mL of ninhydrin solution was added to 5 mL of sample in a series of caped test tubes which were placed at 80 °C in the water-bath with a gentle stirring fixed at 70 rpm. This temperature was chosen in the interval between 80 and 100 °C according to the previous studies with ninhydrin and 2-hydroxynaphthaldehyde reagents. After cooling the reaction tubes at room temperature, the solution absorbance corresponding to 1 h of reaction was measured by using a UV spectrophotometer (Shanghai Metash Instruments Co., Ltd., Shanghai, China). The maximum absorbance wavelength for lysine and Lysine-KCl solutions, across different concentrations (0.1–0.9 mmol L^−1^), was approximately 479 nm. Notably, this maximum absorbance wavelength remained consistent regardless of various experimental parameters such as reaction time, lysine to ninhydrin ratio, reaction temperature, and pH. The optimization of experimental conditions was carried out using a full-factorial design methodology. Hence, potassium, as a representative interfering species, did not interfere with ninhydrin at 479 nm.

### 2.4. Carbohydrate Content Determination

#### 2.4.1. Soluble Sugars

Soluble sugars were extracted and quantified following a previously established method [34]. Briefly, 0.5 g samples were homogenized in 10 mL of deionized water, followed by the addition of 5 mL of 80% ethanol. The mixture was then incubated in a water bath at 45 °C for 20 min. The homogenate was allowed to come to room temperature before undergoing two cycles of centrifugation at 6000 rpm for 10 min at 15 °C. The resulting supernatant was then divided into an aliquot of 2 mL and combined with 2 mL of 3,5-dinitrosalicylic acid reagent, boiling for 5 min, and then allowed to cool in an ice-water bath. The soluble sugar content of the supernatant was quantified at 540 nm using a UV spectrophotometer (Shanghai Metash Instruments Co., Ltd., Shanghai, China). Distilled water was used as a control, and a standard curve utilizing glucose was constructed for reference.

#### 2.4.2. Amylopectin Content

Using the double-wavelength approach, the amylopectin content was spectrophotometrically measured [35]. To determine the starch content, 0.1 g of milled grains were mixed with 10 mL of 0.5 M KOH and heated at 90 °C for 30 min. The mixture was then diluted to 50 mL with distilled water. A 2.5 mL portion of this solution was transferred to a fresh tube containing 20 mL of distilled water. The pH was adjusted to 3.5 using 0.1 M HCl, and 500 μL of I_2_-KI reagent was added to the distilled water, yielding a solution which was further diluted to 50 mL. After standing for 15 min, the absorbance was assessed at 550 nm and 735 nm using a UV spectrophotometer (Shanghai Metash Instruments Co., Ltd., Shanghai, China).

The C/N ratio was calculated with the formula:

C/N ratio = (soluble sugar content + amylopectin content)/total N content.

### 2.5. Determination of Enzyme Activities

#### 2.5.1. Anthocyanin Metabolism Enzyme

The activities of phenylalanine ammonia-lyase (PAL), flavanone 3-hydroxylase (F3H), dihydroflavonol4-reductase (DFR), anthocyanidin synthase (ANS), and UDP-glycose: flavonoid-3-O-glycosyltransferase (UFGT) were measured using specific ELISA kits for each enzyme, namely the plant PAL ELISA kit, plant F3H ELISA kit, plant DFR ELISA kit, plant ANS ELISA kit, and plant UFGT ELISA kit. All kits were used according to the manufacturer’s instructions and were obtained from Enzyme-linked Biotechnology Co., Ltd. (Shanghai, China).

#### 2.5.2. Nitrate Reductase (NR) and Glutamine Synthetase (GS)

Two key nitrogen metabolic enzymes, nitrate reductase (NR) and glutamine synthetase (GS) were selected for detection. NR was extracted and measured using the Nitrate Reductase Assay Kit (BC0080, Solarbio, Beijing, China). Briefly, 0.1 g of fresh seed powder, which had been frozen and ground in liquid nitrogen, was extracted with 1 mL of extraction solution. The mixture was centrifuged at 4000× *g* for 10 min at 4°C, and the supernatant was used to measure the optical density (OD) at 520 nm to calculate the NR activity. The GS activity was assessed using the Glutamine Synthetase Assay Kit (BC0915, Solarbio, Beijing, China). Similarly, 1 mL of extraction buffer was used to extract 0.1 g of fresh seed powder that had been frozen and pulverized in liquid N. After centrifugation at 8000× *g* for 10 min at 4 °C, the supernatant was collected to measure the OD at 520 nm, which was used to calculate the GS activity.

#### 2.5.3. Carbon-Metabolism-Related Enzymes

The preparation procedure was adapted from a previously published method [36]. To assay the enzyme activities, 5–10 g of frozen grains were homogenized in a pre-cooled mortar with 10 mL of ice-cold extraction buffer containing 50 mM Hepes-NaOH (pH 7.5), 2 mM KCl, 5 mM EDTA, 1 mM DTT (dithiothreitol), and 1% (*w*/*v*) PVP (polyvinylpyrrolidone-30). A 30 μL aliquot of the homogenate was diluted with 1.8 mL of extraction buffer and centrifuged at 2000× *g* for 10 min at 4 °C. The supernatant was then further centrifuged at 10,000× *g* for 30 min at 4 °C. The resulting supernatant was used to determine the activities of ADP-glucose pyrophosphorylase (AGPase), soluble starch synthase (SSS), starch branching enzyme (SBE), and a starch debranching enzyme (SDBE). Additionally, 50 μL of the crude enzyme solution was boiled for 60 s prior to analysis. NADH production was measured spectrophotometrically at 340 nm for AGPase and SSS, 660 nm for SBE, and 540 nm for SDBE using a UV spectrophotometer (Shanghai Metash Instruments Co., Ltd., Shanghai, China).

### 2.6. Data Analysis

Statistical analysis was performed using Excel 2016 (Microsoft Office, Washington, USA) and SPSS v.26 (SPSS, Chicago, IL, USA). Specifically, Excel 2016 was used for basic data processing, while SPSS v.26 was employed for advanced statistical analyses, including the one-way analysis of variance (ANOVA) and multiple comparisons using the least significant difference (LSD) test at *p* ≤ 0.05. The normality of the data distribution was assessed using the “nortest” package in RStudio (Version: 2024.04.2+764, https://posit.co/downloads/, accessed on 5 May 2024). Differences in the tested parameters, influenced by the nitrogen application rate, filling stage, year, and their interactions, were examined using a three-factor ANOVA by conducting the “agricolae” package in RStudio. Upon the ANOVA revealing significant differences for any parameter, a least significant difference (LSD) test was conducted for multiple comparisons at *p* ≤ 0.05. The accumulation rate curves were generated utilizing the “ggplot2” package in RStudio. Regression analysis was conducted with both the “ggplot2” and “ggsignif” packages in RStudio. Correlation analysis was carried out with the Origin2022 software (OriginLab Corporation, Northampton, MA, USA). Data in figures were the average of three biological replicates.

## 3. Results

### 3.1. Analysis of Variances (ANOVA)

The results of the ANOVA demonstrated that nearly all investigated traits were significantly influenced by the nitrogen level (NL), with the exception of phenylalanine ammonia-lyase activity (PALA). Similarly, the filling stage (FS) significantly affected all traits except for flavanone 3-hydroxylase activity (F3HA), UDP-glycose flavonoid-3-O-glycosyltransferase activity (UFGTA). Additionally, several traits exhibited significant variation due to year (Y). Notably, the interactions among these factors, including Y × NL, Y × FS, NL × FS, and Y × NL × FS, were significant for only a few traits (Table 1). Among these factors, NL exerted the most substantial effect on all traits except for phenylalanine ammonia-lyase activity (PALA) and C/N ratio, underscoring the pronounced responsiveness of the investigated traits to the nitrogen application rate (Table 1).

### 3.2. Dynamic Changes of Anthocyanin Biosynthesis in Grains of Purple Waxy Maize under Different N Application Rates

#### 3.2.1. The Seed Color and Anthocyanin Accumulation

**Figure 1:**
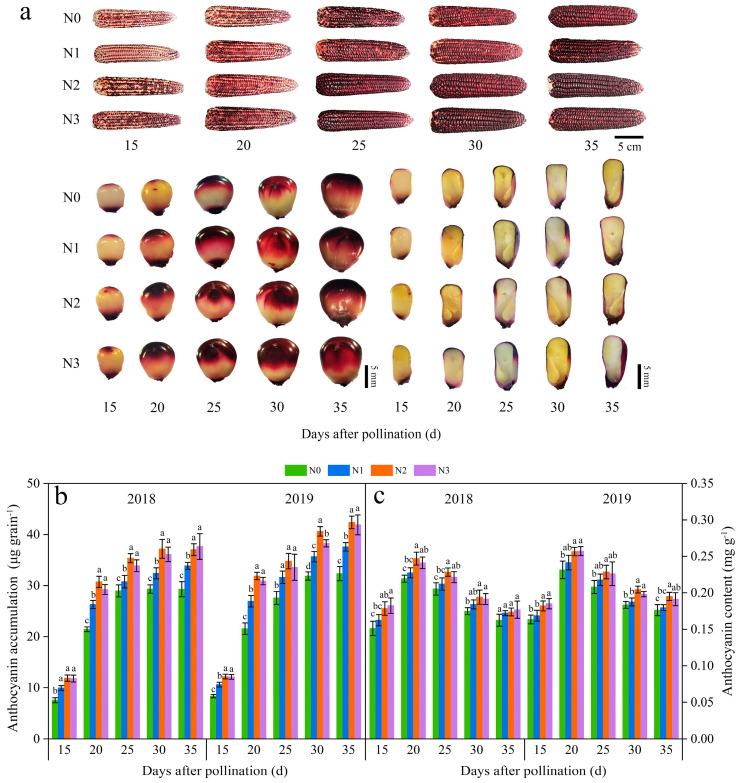
Effects of N application doses on dynamic changes in anthocyanin accumulation amount (AAA) and content in grains of purple waxy maize at different days after pollination. (**a**) dynamic changes of the phenotypes of Jinnuo20 ears and kernels; (**b**) anthocyanin accumulation amount (AAA); (**c**) anthocyanin content (ANC). Different lowercase letters denote significant differences between N rates at the *p* < 0.05 level. The error bar denotes standard error based on 3 data.

The anthocyanin accumulation amounts (AAAs) in JN20 kernels rose progressively from 15 to 30 days after pollination (DAP) and essentially halted, accumulating at 30 DAP under all four nitrogen (N) treatments (Figure 1a). Compared to the N0 treatment, the other three N application treatments expedited the rise in AAA at five different filling time periods (Figure 1b and Figure S1). Among these treatments, AAA under 240 kg N ha⁻¹ (N2) was considerably greater than that under 120 kg N·ha⁻¹ (N1) and slightly higher than that under 360 kg N ha⁻¹ (N3) in both 2018 and 2019, indicating that the N application had a notable effect on anthocyanin accumulation in purple waxy corn grains. Overall, the effects of the N application rate on anthocyanin accumulation adhered to the hierarchy N2 ≥ N3 > N1 > N0. Specifically, the AAA in a single grain at 20, 25, 30, and 35 DAP increased by averages of 161.21%, 209.45%, 238.58%, and 250.63%, respectively, in comparison to 15 DAP across the four N application rates over two years. Additionally, AAA exhibited average increases of 18.34%, 35.62%, and 32.35% under N1, N2, and N3, respectively, compared to N0 across the five filling time points during a two-year period (Figure 1b). The peak accumulation rate was observed around 20 DAP under all four N levels in both years (Figure S2).

Further analysis revealed that the anthocyanin content (ANC) in JN20 grains exhibited analogous trends, with the hierarchy of N2 ≥ N3 > N1 > N0 observed across five filling stages over two years (Figure 1c and Figure S1). The average increases in ANC under the N1, N2, and N3 treatments were 4.35%, 11.49%, and 10.81%, respectively, compared to the N0 treatment (Figure 1c). In contrast, ANC in grains rose from 15 DAP to 20 DAP and then progressively declined from 20 DAP to 35 DAP in both 2018 and 2019 (Figure 1c and Figure S1). In addition, ANC in grains revealed average increases of 40.28%, 26.50%, 10.84%, and 4.29% at 20, 25, 30, and 35 DAP, respectively, in comparison to 15 DAP across the four N levels over a two-year period (Figure 1c).

#### 3.2.2. The Anthocyanin-Biosynthesis-Related Enzymes

Given that anthocyanin accumulation in JN20 kernels increased rapidly from 15 to 20 days after pollination (DAP) and nearly ceased from 30 to 35 DAP, the activities of five key anthocyanin-biosynthesis-related enzymes, including phenylalanine ammonia-lyase (PAL), dihydroflavonol 4-reductase (DFR), flavanone 3-hydroxylase (F3H), anthocyanidin synthase (ANS), and UDP-glycose: flavonoid 3-O-glucosyltransferase (UFGT) were subsequently measured in fresh JN20 grains at 15 and 30 DAP. According to Table 2, the activities of PAL, F3H, and ANS at 15 DAP all increased first and then decreased with the increase in N application rates, and reached the peak values under N2 or N1 treatment. In detail, PAL, F3H, and ANS activity exhibited significant increases under N1, N2, and N3 treatments compared to N0 treatment, with enhancements of 12.10–48.92%, 2.71–34.61%, and 0.09–28.99% throughout both years, respectively. On the contrary, the activities of PAL, F3H, and ANS at 30 DAP gradually diminished with escalating N application rates, and the reductions ranging from 4.93% to 27.27%, 7.72% to 14.71% and 2.47% to 19.26% under N1, N2, and N3 treatments, compared to N0 treatment over two years, respectively.

Additionally, the UFGT activity reached the peak values under N2 or N3 treatment at 15 DAP and under N0 or N1 treatment at 30 DAP over the two-year period, with an average change range of −10.57% to 30.25% under N1, N2, and N3 treatments compared to the N0 treatment (Table 2). However, the variations in DFR activity among different N application treatments over the two years were more intricate. In 2018, the DFR activity was higher under N0 and N1 treatments compared to N2 and N3 treatments, whereas in 2019, there were no significant differences among the various N application rates (Table 2). These results indicate that the N treatment enhances the activities of PAL, F3H, and ANS in purple waxy corn grains during the early filling stage, while inhibiting their activities during the later filling stage.

#### 3.2.3. The Regression Analysis

**Figure 2:**
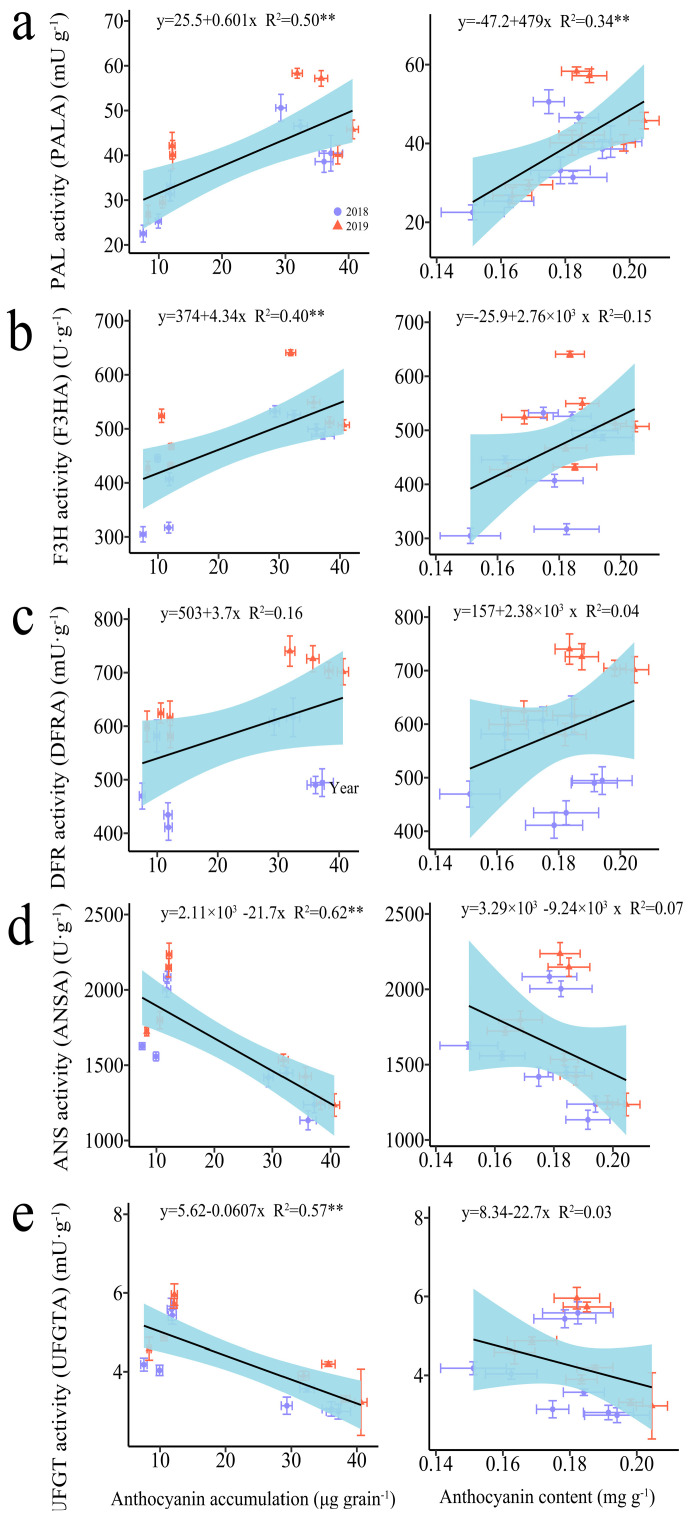
Relationship between anthocyanin accumulation amount (AAA), anthocyanin content (ANC), and anthocyanin-biosynthesis-related enzymes in grains of purple waxy maize on different days after pollination. ** signify significant differences at the *p* < 0.01 levels.

The regression analysis indicated a significant positive correlation between anthocyanin accumulation amounts (AAA) and the activities of phenylalanine ammonia-lyase (PAL) and flavanone 3-hydroxylase (F3H), whereas significant negative correlations were observed with the activities of anthocyanidin synthase (ANS) and UDP-glucose flavonoid 3-O-glucosyltransferase (UFGT) (Figure 2). In contrast, the anthocyanin content (AC) was only found to be significantly positively correlated with PAL activity (Figure 2).

### 3.3. Dynamic Changes in N Metabolism in Grains of Purple Waxy Maize under Different N Application Rates

#### 3.3.1. The Total N Content (TNC)

**Figure 3:**
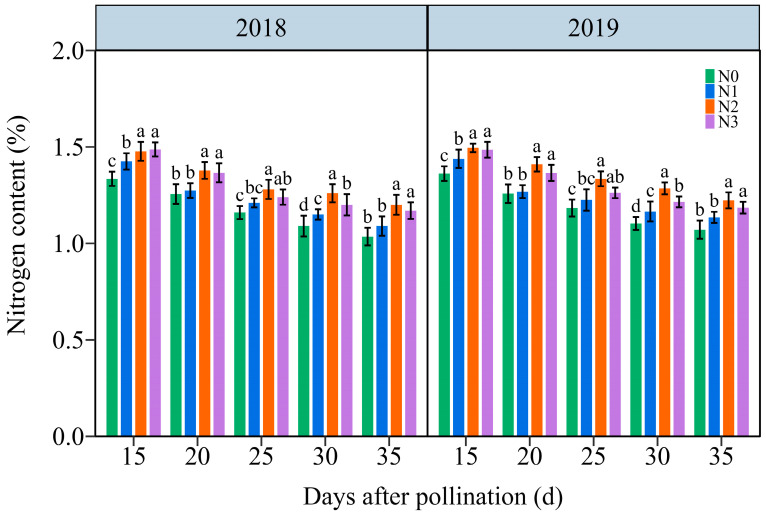
Effects of N application doses on the dynamic changes in the kernel N content in grains of purple waxy maize on different days after pollination. Different lowercase letters denote significant differences between N rates at the *p* < 0.05 level. The error bar denotes standard error based on 3 data.

Under the four N application treatments, the TNC in JN20 grains exhibited a consistent decline throughout the filling stages over time (Figure 3 and Figure S3). The effects of the N application on TNC in waxy maize grains also exhibited N2 ≥ N3 > N1 > N0 at the five filling times in two years. The TNC under N1, N2, and N3 treatments increased significantly in the ranges of 0.26–14.46%, 4.22–21.58%, and 1.82–14.67% compared to N0, respectively (Figure 3). However, the grain TNC at 20, 25, 30, and 35 DAP diminished progressively by an average of 8.03%, 13.96%, 17.72%, and 20.84%, respectively, compared to 15 DAP according to the four N application rates within both years. It reached a peak accumulation rate around 20 DAP under four N levels within both years (Figure S4). These results illustrated that the N application had certain effects on N transport and assimilation in purple waxy corn grains.

#### 3.3.2. The Nitrite Content (NC)

**Figure 4:**
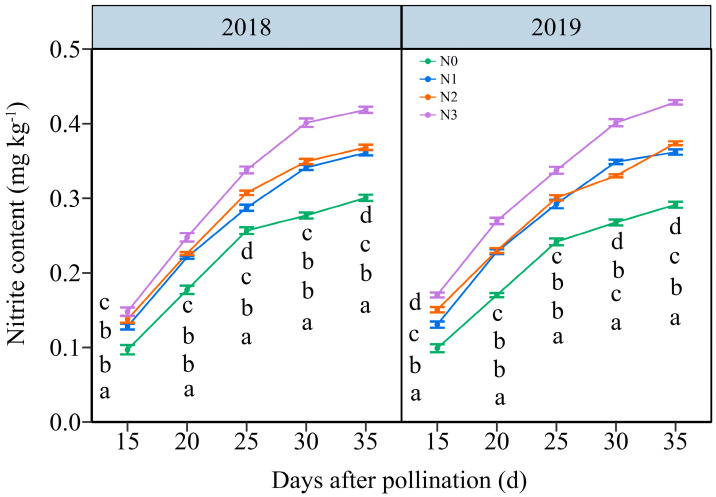
Effects of N application doses on dynamic changes in nitrite content (NC) in fresh grains of purple waxy maize at different days after pollination. Different lowercase letters denote significant differences between N rates at the *p* < 0.05 level. The error bar denotes a standard error based on 3 data.

Under the four N application treatments, the NC in JN20 grains represented a similar variation tendency during filling stages, which increased continuously over time (Figure 4 and Figure S5). The effects of N application on NC in grains demonstrated a hierarchy of N3 > N2 ≥ N1 > N0 over the five time points within two years (Figure 4 and Figure S5). The NC under N1, N2, and N3 treatments shown significant increases of 25.42%, 30.20%, and 47.63% compared to N0 over five time points within both years, respectively (Figure 4). However, the grain NC at 20, 25, 30, and 35 DAP gradually increased by an average of 69.73%, 128.99%, 163.27%, and 179.45%, respectively, compared to 15 DAP according to the four N application rates in two years. These results indicated that an accurate N application rate had certain effects on maintaining the nitrite metabolism balance in purple waxy corn grains.

#### 3.3.3. The Lysine Content (LC)

**Figure 5:**
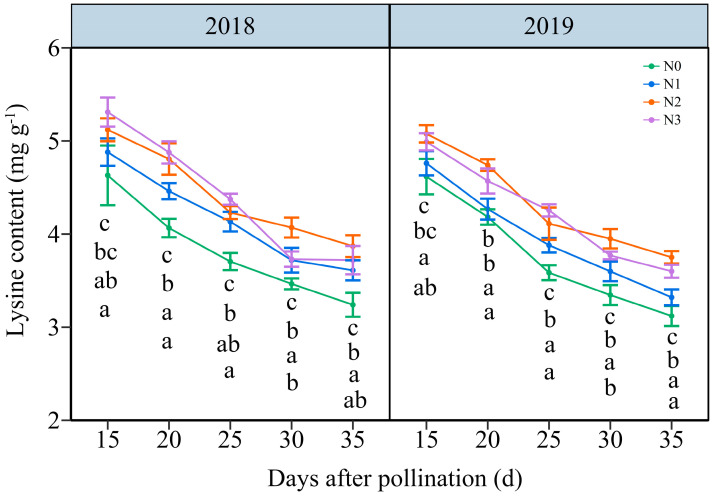
Effects of N application doses on the dynamic changes in lysine content (LC) in fresh grains of purple waxy maize at different days after pollination. Different lowercase letters denote significant differences between N rates at the *p* < 0.05 level. The error bar denotes standard error based on 3 data.

In contrast with NC, the LC in JN20 grains all decreased gradually under the four N levels over time (Figure 5 and Figure S6). The effects of N application on LC in waxy maize grains followed the order N2 ≥ N3 > N1 > N0 across the five grain-filling periods over the two years. The LC under N1, N2, and N3 treatments showed a significant increase, with average ranges of 0.34–13.66%, 4.23–22.36% and 4.00–25.23% compared to N0, respectively (Figure 5). Nevertheless, based on the four N application rates in both years, the LC fell by an average of 8.44%, 17.65%, 24.60%, and 27.80%, respectively, compared to 15 DAP according to the four N application rates within both years. And, it reached a peak accumulation rate of around 20 DAP under four N levels within both years (Figure S7). These results indicated that a reasonable N application rate had certain effects on inducing lysine biosynthesis in purple waxy corn grains.

#### 3.3.4. Nitrate Reductase (NR) and Glutamine Synthetase (GS) Activity

**Figure 6:**
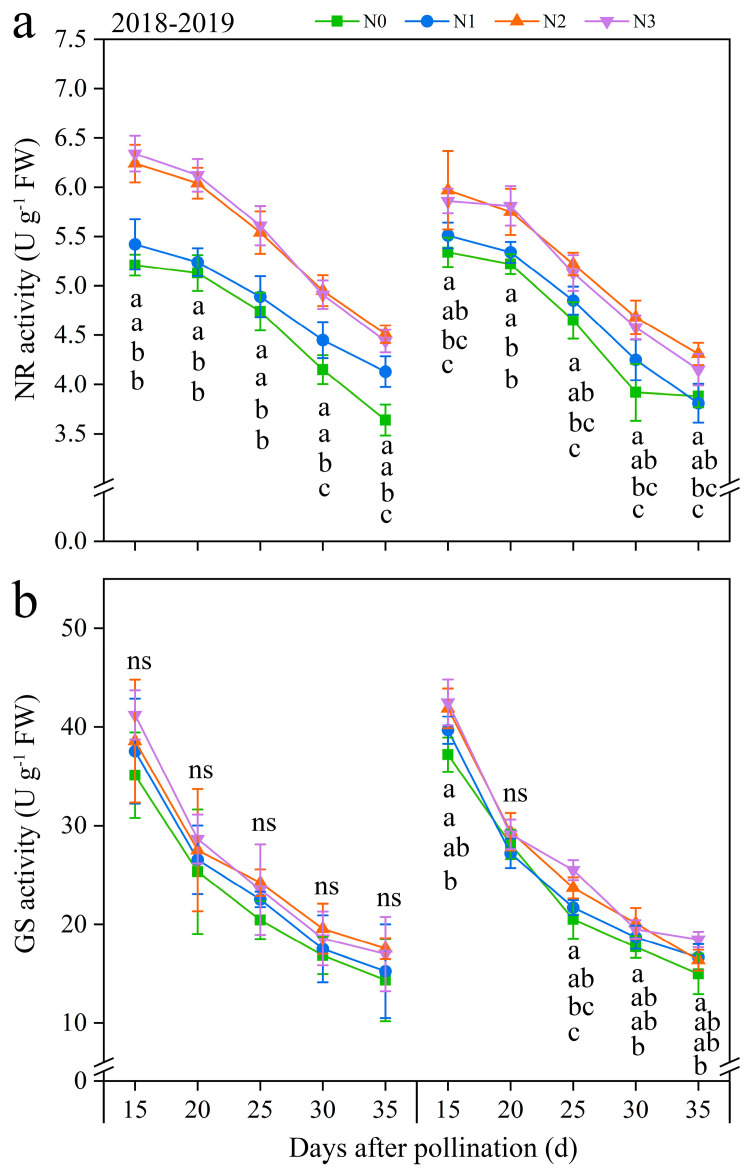
Effects of N application doses on dynamic changes in nitrate reductase (NR) and glutamine synthetase (GS) activities in fresh grains of purple waxy maize at different days after pollination. ns denotes no significant difference. Different lowercase letters denote significant differences between the N rates at the *p* < 0.05 level. The error bar denotes standard error based on 3 data.

Two critical N metabolism enzymes (NR and GS) were identified to further investigate the effects of N application on N metabolism (Figure 6 and Figure S8). Under the four N application treatments, the NR and GS activities exhibited a similar trend of fluctuation during filling periods, with both continuously decreasing over time. Both NR and GS activities were positively modulated by the rate of N application. At the same time, the evolving patterns of N application in NR and GS activities roughly showed N2 ≥ N3 > N1 ≥ N0 at every time point in both years. In detail, compared with N0, the activities of NR and GS under N1, N2, and N3 exhibited significant increases, ranging from 3.26 to 19.90% and from 2.30 to 15.06% in both years, respectively (Figure 6). And, the NR activity at 20, 25, 30, and 35 DAP diminished consistently by an average of 2.70%, 11.46%, 21.79%, and 28.37%, while the GS activity exhibited a gradual decline of 29.29%, 41.97%, 52.68%, and 58.38% decreased, respectively, compared to 15 DAP, combining all N treatments in both years (Figure 6). These results revealed that, while NR and GS activities declined over time, the N application consistently enhanced these activities during the grain-filling stage in purple waxy maize grains.

#### 3.3.5. The Regression Analysis

**Figure 7:**
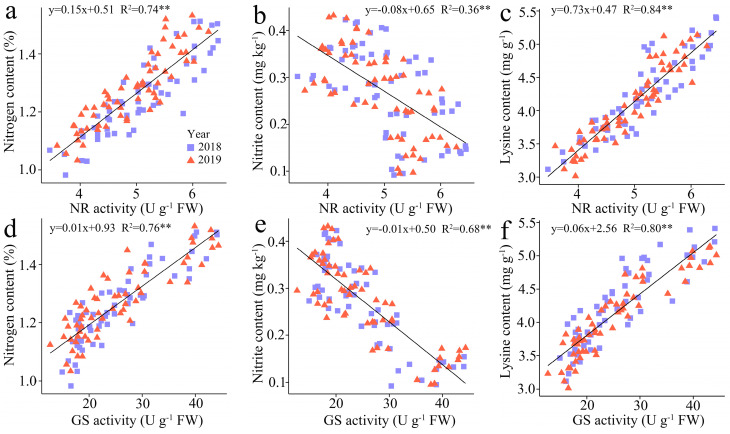
Relationship between the total N content (TNC), nitrite content (NC) , and lysine content (LC) in fresh grains of purple waxy maize at different days after pollination. ** signify significant differences at *p* < 0.01 levels.

The regression analysis indicates a substantial positive correlation between TNC and LC in kernels with the NR activity (R^2^ = 0.74 and 0.84) and GS activity (R^2^ = 0.76 and 0.80), but the NC in kernels showed a negative correlation with NR and GS activity, and the R^2^ of which was 0.36 and 0.68, respectively (Figure 7).

### 3.4. Dynamic Changes in Carbohydrate Biosynthesis in Grains of Purple Waxy Maize under Different N Application Rates

#### 3.4.1. The Soluble Sugar Content (SSC)

**Figure 8:**
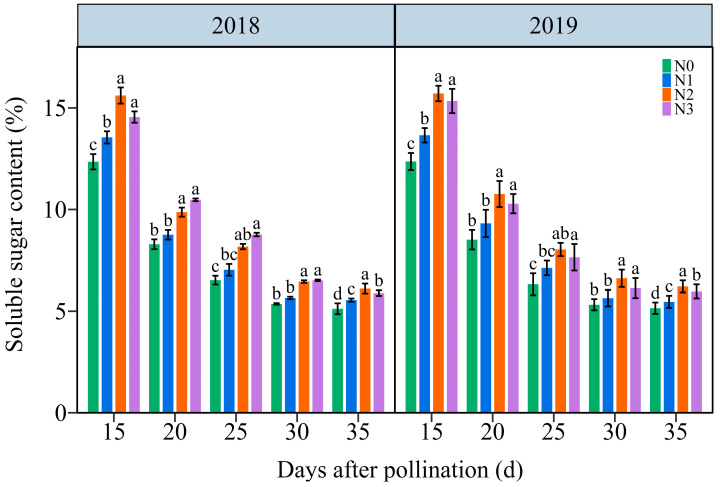
Effects of N application doses on dynamic changes of soluble sugar content (SSC) in fresh grains of purple waxy maize on different days after pollination. Different lowercase letters denote significant differences between the N rates at the *p* < 0.05 level. The error bar denotes standard error based on 3 data.

The SSC in JN20 grains showed a similar variation tendency during filling stages, continuously declining under all four N application treatments over time (Figure 8 and Figure S9). The effects of N application on SSC in waxy maize grains followed the order N2 ≥ N3 > N1 > N0 at the five filling times in two years. The SSC significantly increased under the N1, N2, and N3 treatments, with ranges of 1.03–26.54%, 9.37–38.81%, and 7.72–40.12% compared to N0, respectively (Figure 8). However, the SSC at 20, 25, 30, and 35 DAP gradually diminished by an average of 32.53%, 47.13%, 58.39%, and 60.06%, respectively, relatively to 15 DAP according to the four N application treatments in both years (Figure 8). These results suggested that a precise N application rate had certain effects on soluble sugar biosynthesis in purple waxy corn grains.

#### 3.4.2. The Amylopectin Content (AC)

**Figure 9:**
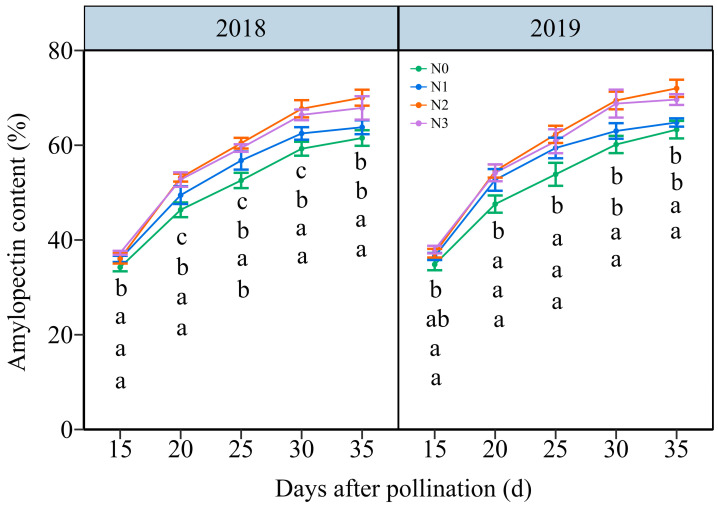
Effects of N application doses on dynamic changes in amylopectin content (AC) in the fresh grains of purple waxy maize on different days after pollination. Different lowercase letters denote significant differences between the N rates at the *p* < 0.05 level. The error bar denotes a standard error based on 3 data.

As shown in Figure 9 and Figure S10, the AC in grain increased with time, showing an average increase from 36.27% to 66.62% from 15 DAP to 35 DAP, respectively. The effects of the N application on AC in waxy maize grains demonstrated a hierarchy of N2 ≥ N3 > N1 > N0 over five filling periods in two years. In detail, the grain AC rose from an average of 34.54%, 26.26%, 36.66%, and 37.63% at 15 DAP to 62.40%, 63.30%, 71.02%, and 68.76% at 35 DAP under N0, N1, N2, and N3 within two years, respectively. And, it reached a peak accumulation rate of around 20 DAP under four N levels within both years (Figure S11). These results indicated that augmenting the N dose greatly enhanced the amylopectin biosynthesis in waxy maize grains.

#### 3.4.3. Carbohydrate-Metabolism-Related Enzymes

**Figure 10:**
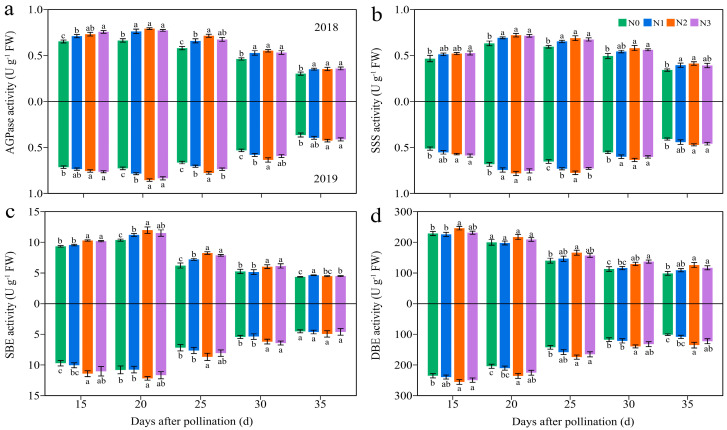
Effects of the N rate on the enzymatic activity of carbon metabolism in fresh grains of purple waxy maize at different days after pollination. Different lowercase letters denote significant differences between N rates at the *p* < 0.05 level. The error bar denotes standard error based on 3 data.

Four C metabolism enzymes in JN20 grains, namely soluble starch synthase (SSS), starch branching enzyme (SBE), and starch debranching enzyme (SDBE) participating in amylopectin synthesis, and ADP-glucose pyrophosphorylase (AGPase) for sugar metabolism, were determined. The activities of AGPase and SSS exhibited significant variations solely among the N1, N2, and N3 treatments in 2019 (Figure 10 and Figure S12). Over time, the activity of DBE declined steadily, while the activities of AGPase, SSS, and SBE increased from 15 to 20 DAP before gradually declining. Furthermore, the application of N enhanced the activities of the AGPase, SSS, and SBE activity (Figure 10 and Figure S12). The impacts of N application on DBE activity were relatively complex. The N effects for DBE activity was not significant from 15 to 25 DAP in 2018, but the DBE activity increased significantly and stabilized from 25 to 35 DAP under N2 and N3 treatments (Figure 10 and Figure S12). Meanwhile, N application had a significant impact on DBE activity in 2019. For example, the AGPase activity increased by 1.91–20.85%, 4.36–26.82%, and 1.29–29.68% under N1, N2, and N3 treatments compared to N0 over the five grain filling time points over two years, respectively (Figure 10).

#### 3.4.4. The Regression Analysis

**Figure 11:**
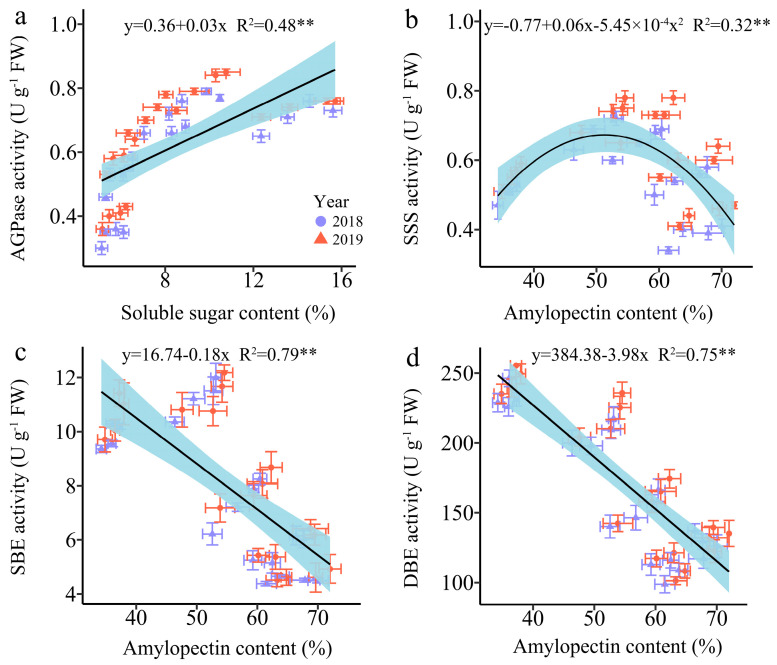
Relationship between soluble sugar content (SSC), amylopectin content (AC) and the enzymatic activity of the carbon metabolism in fresh grains of purple waxy maize on different days after pollination. ** signify significant differences at the *p* < 0.01 levels.

Since SSS, SBE, and SDBE are involved in amylopectin synthesis, and AGPaseis is responsible for converting soluble sugar into starch [18]. Therefore, we further examined the linear relationship between the relevant enzymes and carbohydrates (Figure 11). The analysis revealed a highly significant positive correlation between AGPase activity and SSC, while the SBE and DBE activity were found to have a strong significant negative correlation with AC. Furthermore, the relationship between SSS activity and AC was a univariate quadratic correlation, indicating a positive correlation between SSS activity at low AC and a negative correlation at high AC (Figure 11).

### 3.5. Effects of the N Levels on the Dynamic C/N Ratio in Grains of Purple Waxy Maize

**Figure 12:**
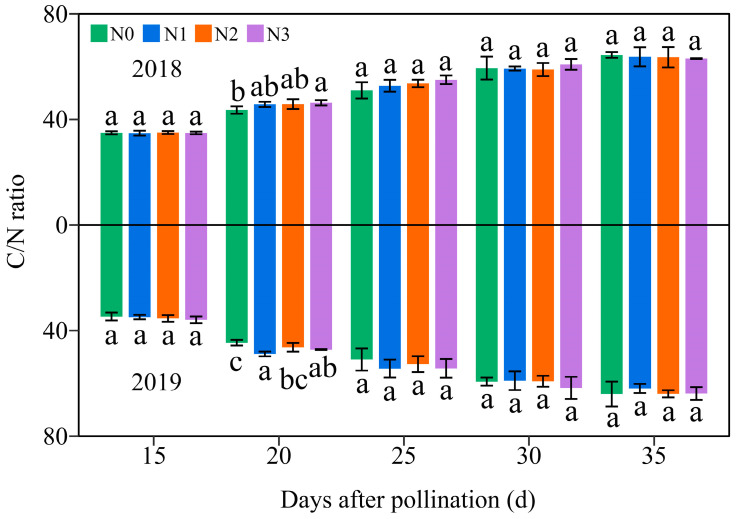
Effects of the N rate on the grain C/N ratio in fresh grains of purple waxy maize on different days after pollination. Different lowercase letters denote significant differences between N rates at the *p* < 0.05 level. The error bar denotes standard error based on 3 data.

Over the two-year period, the C/N ratio in JN20 grains gradually increased across the four N levels (Figure 12 and Figure S13), suggesting that the N accumulation in waxy corn grains occurs earlier than the C accumulation. Comparatively, C/N ratio at 20, 25, 30, and 35 DAP was induced by an average of 31.38%, 51.46%, 70.33%, and 81.35% relative to 15 DAP, respectively, over the four N treatments within two years (Figure 12). In addition, it showed that applying N had a slight effect on the C/N ratio at 20 DAP, but it caused no significant effect in the C/N ratio during the whole grain filling stages (15–35 DAP) in general (Figure 12). Hence, it was confirmed that carbohydrates accumulated prior to N. In detail, the C/N ratio under N1, N2, and N3 treatments grew by 0.31–13.02%, 0.83–11.52%, and 1.37–14.80% compared to N0 over the five grain filling stages within the two years, respectively (Figure 12).

### 3.6. Correlation Analysis

**Figure 13:**
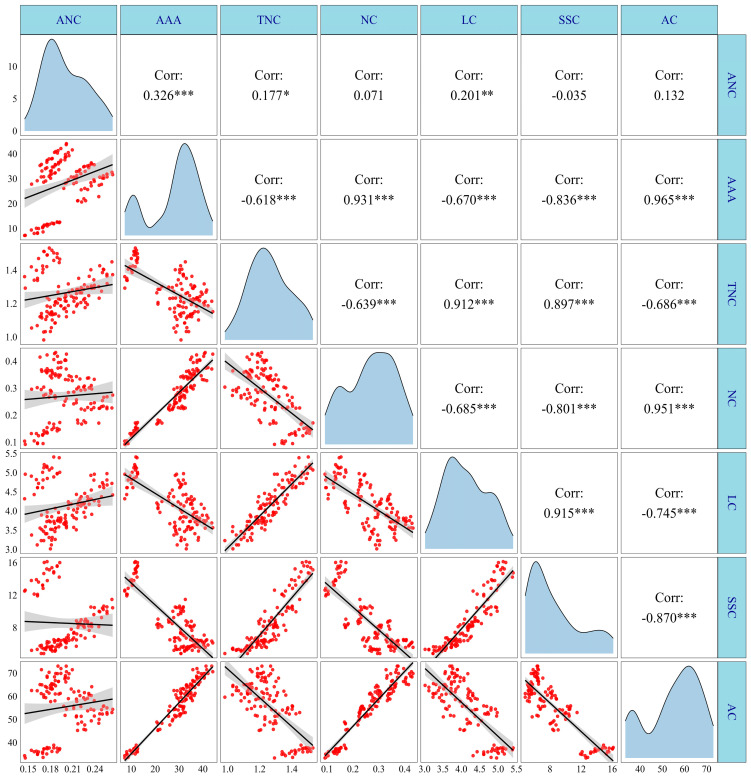
Correlation analysis of anthocyanin, N metabolism, and carbohydrate content of purple waxy maize. *, ** and *** denote significant differences at the *p* < 0.05, *p* < 0.01 and *p* < 0.001 levels, respectively.

As shown in Figure 13, there were significant positive correlations between the anthocyanin content (ANC) and the anthocyanin accumulation amount (AAA) and lysine content (LC); AAA positively correlated with ANC and nitrite content (NC); total nitrogen content (TNC) positively correlated with the soluble sugar content (SSC) and LC; NC had significant positive correlations with AC, LC, and SSC. In contrast, there were significant negative correlations between AAA and LC and TNC; TNC was significantly negatively correlated with AC and NC; NC was negatively correlated with and SSC and LC; LC had significant negative correlations with AC, SSC, and AC. It was noted that there were no significant correlations between ANC and LC, ANC and SSC, and ANC and TNC, but these were clustered into two distinct regions following the regression analysis. Therefore, further segmented regression analysis was conducted, and the analysis results revealed that there were significant positive correlations between ANC and LC, ANC and SSC, and ANC and TNC at 15 DAP and during the period of 20–35 DAP, with an R^2^ of 0.38, 0.37, 0.48, at 15 DAP and 0.72, 0.37, and 0.68 during the period of 20–35 DAP, respectively (Figure S14).

## 4. Discussion

### 4.1. The Appropriate N Application Rate Promotes Nitrogen Metabolism in Waxy Maize Kernels

Nitrogen (N) plays a crucial role as a vital constituent in numerous plant physiological processes, encompassing enzyme activities, hormonal regulation, and amino acid biosynthesis [37]. A recent study demonstrated that the kernel N content was significantly increased when subjected to applications of 84, 168, and 224 kg N ha^−1^, as compared to plots where no N was applied. Moreover, the advantageous impact of N fertilization on accumulation demonstrated a positive correlation with escalating application rates [38]. Similarly, a study conducted in rice and maize revealed that the N content in grains exhibited an upward trend in response to incremental N application rates ranging from 0 to 240 kg N ha^−1^, applied at intervals of 40 kg N ha^−1^ [39]. In contrast, multiple studies have illustrated that the influence of N application on grain quality is substantially reliant on dosage. Appropriate fertilization practices have been identified as one of the most efficacious strategies for enhancing maize quality, but excessive N application has been shown to have detrimental effects on grain quality [40,41,42]. This study revealed that N doses significantly enhance the kernel N content, with 240 kg N ha^−1^ attaining the highest kernel N content, exhibiting an increase range of 4.22–21.58% compared to 0 kg N ha^−1^, indicating that optimal N application was necessary to stabilize the kernel N content. Concurrently, the kernel N content consistently diminished, averaging between 8.03% and 20.8% from 15 DAP to 35 DAP under four N treatments, which indicates that the accumulation and transport of carbohydrates in the grains should take precedence over N accumulation [43].

Nitrate reductase (NR) is the first enzyme in the pathway that converts nitrate into nitrite, hence restricting the total N assimilation in plants [43]. Glutamine synthetase (GS) catalyzes the ATP-dependent conversion of glutamate and ammonia into glutamine, providing substrate for amino acid synthesis, and acts at the center of N metabolism [44]. The current study revealed that the effects of N doses on NR and GS activities paralleled those on kernel N content, and it showed that NR and GS activities both peaked at the N2 treatment compared to the three other N treatments, and the regression analysis demonstrated a significant positive correlation between NR and GS activity and the kernel N content, illustrating that an appropriate N application rate is crucial for enhancing in NR and GS activities during the filling phases and ensuring the N metabolism.

Excessive levels of nitrite in cereals can pose health risks to consumers, as nitrite has been associated with adverse health effects such as methemoglobinemia and potential carcinogenicity [45]. With regard to consumer safety, consuming a single serving of a nitrate-rich food or supplement may exceed the World Health Organization’s recommended daily intake limit for nitrate, which ranges from 0 to 3.7 mg per kilogram of body weight per day, or 222 mg per day for a 60 kg adult [46]. Hence, it is crucial for food producers and regulators to monitor and adhere to these safety standards to protect public health and ensure the quality of cereal products in the market. Lysine is an essential amino acid that plays a crucial role in protein synthesis and overall human health [47]. Maize is known to be deficient in lysine compared to other cereal grains, which can impact the nutritional quality of maize-based diets [48]. Hence, increasing lysine levels in maize kernels can contribute to better overall nutrition and health outcomes, especially in regions with high maize consumption rates. In our study, we found that the nitrite content (NC) in waxy maize grains gradually increased during filling stages. Notably, the NC in the kernels reached 0.32 mg kg^−1^ under the 360 kg N ha^−1^ treatment, which was significantly higher than that under the 0 kg N ha^−1^, 120 kg N ha^−1^, and 240 kg N ha^−1^ treatments over two years. In contrast, the NCs under N1 and N2 were relatively similar, at 0.27 mg kg^−1^ and 0.28 mg kg^−1^ over two years. Although the maximum value was about 0.5 mg kg^−1^, which is still a gap from the maximum safety standard value, we propose that an appropriate N application rate still has great benefits in reducing the risk of the excessive consumption of nitrite. Furthermore, the lysine content in waxy maize grains decreased continuously during filling stages, and was induced much greater under 360 kg N ha^−1^ and 240 kg N ha^−1^ than 120 kg N ha^−1^ compared to 0 kg N ha^−1^, and the values of which were relatively close under 240 kg N ha^−1^ and 360 kg N ha^−1^. Hence, for lysine and nitrite, under a reasonable N application dose, like 240 kg N ha^−1^, the waxy maize should be harvested early and in a timely manner when it is edible to ensure that more lysine and less nitrite are consumed. In addition, the regression analysis indicated that NR and GS activity had a strong positive association with the lysine content, while demonstrating negative correlations with nitrite content. Consequently, we suggest that the judicious use of N can promote activities of N metabolism enzymes, thereby mitigating nitrite accumulation and augmenting lysine synthesis in waxy maize kernels.

### 4.2. The Reasonable Application of N Induces Carbohydrate Biosynthesis

N serves as the predominant element in chlorophyll, playing a pivotal role in influencing leaf biomass accumulation and photosynthetic efficiency [49], and carbohydrates are almost synthesized in plants through photosynthesis. Numerous studies have investigated the effects of N fertilization on carbohydrate accumulation in maize kernels, demonstrating that increased N application during the grain filling phase promotes the accumulation of starch and soluble sugars in maize kernels [50,51]. N fertilizer has been shown to enhance starch accumulation in wheat, and higher amylopectin and total starch contents were obtained when the N level was 240 kg ha^−1^ [52]. Conversely, a prior study demonstrated a gradual reduction in amylose, amylopectin, and total starch contents in wheat with a rising N content, but the rate of starch accumulation exhibited a marked increase under these conditions [53]. The study demonstrates that the N level significantly influences the soluble sugar and amylopectin contents at any grain filling stage within two years, consistently exhibiting the order 240 kg N ha^−1^ ≥ 360 kg N ha^−1^ > 120 kg N ha^−1^ > 0 kg N ha^−1^. The soluble sugar and amylopectin content in the grains of JN20 under 240 kg N ha^−1^, for which might be the most reasonable dose in this study, increased by an average of 23.74% and 13.00% compared to 0 kg N ha^−1^, over the five grain filling stages within two years. Meanwhile, this study showed that applying N generally had no significant effects on the C/N ratio in waxy maize grains during the whole grain-filling stages.

In general, ADP-glucose pyrophosphorylase (AGPase), starch debranching enzyme (DBE), soluble starch synthase (SSS), and starch branching enzyme (SBE) are essential enzymes that significantly influence sugar and starch biosynthesis, regulating the complex process of starch synthesis and subsequent accumulation [54]. Studies have indicated that the N availability directly impacts the activities of enzymes involved in carbon (C) metabolism, thereby affecting C accumulation in plant tissues [55]. Numerous studies had reported that the activity of the major C metabolic enzyme in wheat, rice, and potato as influenced by applying N fertilizer. The starch accumulation rate was higher at higher N levels, and the activity of SSS in wheat was substantially enhanced by the N application [56]. The activities of AGPase, SSS, and SBE in rice were dramatically elevated following the N supply [57]. In contrast, the activities of the AGPase and SSS in potato markedly increased under a low N level and subsequently declined with an increasing N level, while the SBE activity exhibited no significant response to N fertilization [58]. This study identified dynamic alterations in the activities of AGPase, SSS, SBE, and SDBE in the grains of purple waxy maize variety JN20 after applying different N rates. Our findings revealed that N application increased the AGPase and SSS activity, while the effects on SBE and SDBE activity were not significant, and the activities of AGPase and SSS were maintained at a higher level compared to other N application treatments. The regression analysis results demonstrated a strong positive correlation between AGPase activity and soluble sugar, while the SBE and DBE activity exhibited a substantial negative correlation with the amylopectin content. In conclusion, we postulated that appropriately applying N can regulate the activities of related C metabolism enzymes to induce C biosynthesis in waxy maize kernels.

### 4.3. The Reasonable Application of N Promotes Anthocyanin Biosynthesis in the Kernels of Purple Waxy Maize

Anthocyanins are crucial for protecting plants from ultraviolet radiation, enticing pollinators and having antioxidant effects [59]. Besides their intrinsic quality, the color gradation of purple waxy corn is also an economic characteristic. The elevated anthocyanin content in waxy maize kernels positively influences both shell coloration and human health. Previous studies have demonstrated that N deficiency can have an increase in anthocyanin content in *Arabidopsis* leaves [60], apple seedlings [61], grapes [62], red lettuce [63], and radish [26]. However, two recent studies on purple rice found that N application promotes the accumulation of anthocyanins in leaf sheaths [27], and in the grains of specific purple rice genotype [64]. Furthermore, these findings indicate that the regulation of N in anthocyanin synthesis is highly intricate and may depend on specific plant tissues or organs. The present investigation indicates that the anthocyanin accumulation and content in grains were more significantly influenced by an elevated N application and 240 kg N ha^−1^ compared to the three other N treatments.

Generally, N deficiency typically elevates the expression level of anthocyanin-synthesis-related enzymes in plants, hence modulating anthocyanin accumulation. In *Arabidopsis*, *PAL*, *F3H*, *ANS*, and *DFR* are upregulated in leaves during N deficiency [60]. In tobacco, PAL activity in its leaves and roots increases with N deficiency condition [65]. This study found that the activities of PAL, ANS, and UFGT at 15 DAP were enhanced by increasing the N application rate, with all showing their highest values under 240 kg N ha^−1^, but were suppressed at 30 DAP. Regression and correlation analysis results showed that the anthocyanin accumulation was positively correlated with PAL, F3H, and DFR activity and negatively correlated with ANS and UFGT activities. The anthocyanin content was positively correlated with the PAL, F3H and DFR activity and negatively correlated with ANS activity. These findings indicate that the N application regulates N metabolism in the grains, thereby modulating the activities of the anthocyanin biosynthesis enzymes, resulting in an increasing accumulation of anthocyanins in the grains of purple waxy corn, of which PAL, F3H and DFR might play an important role in the accumulation of anthocyanins, and ANS and UFGT might participate in anthocyanin homeostasis in the grains purple waxy corn. Primarily, these results unequivocally indicate that a precise N application rate contributes to modulating the activities of PAL, ANS, and UFGT in the grains of waxy corn, hence enhancing anthocyanin biosynthesis.

Research has shown that carbohydrates increase anthocyanin biosynthesis in several plant species [66]. Sugars are the precursors of anthocyanin synthesis and signal molecules that regulate anthocyanin synthesis [67]. For instance, anthocyanins are stimulated by sugars in cell suspensions of *Vitis vinifera* [68], and in the hypocotyls of radish (*Raphanus sativus*) [69]. Sugars, such as sucrose, glucose, and fructose, all participate in inducing anthocyanin accumulation, but sucrose is the most effective [66]. In addition, anthocyanin-biosynthesis-related genes were significantly induced by sucrose in radish hypocotyl [69] and *Arabidopsis* seedlings [67]. A recent study indicated that the sucrose concentration in maize grains at N application rates of 200 and 300 kg N ha^−1^ exceeds that at 0 and 100 kg N ha^−1^, peaking at 14 days after pollination, suggesting that the optimal nitrogen application enhances the sucrose accumulation during the initial phases of grain filling [37]. In the current study, we demonstrated that carbohydrates were transported to the grains of purple waxy corn prior to N application, with an increased N rate during the early grain filling stages promoting the synthesis and accumulation of sugars in the grains. This process induced the expression of enzymes related to anthocyanin synthesis and regulated the anthocyanin accumulation and content.

## 5. Conclusions

During the filling stages, the contents of anthocyanin, lysine, and soluble sugars in purple waxy maize variety JN20 continued to decrease, while the accumulation of anthocyanin, total N, nitrite content, and amylopectin content increased steadily. The optimal N application rate, such as 240 kg N ha^−1^, stimulated and maintained the activities of nitrate reductase (NR) and glutamine synthetase (GS) in the grain of waxy corn, regulating grain N metabolism to accumulate more N, relatively less nitrite, and increased lysine. Additionally, this enhanced the activities of enzymes related to sucrose (AGPase) and amylopectin biosynthesis (SSS, SBE, and SDBE), effectively modulating the biosynthesis and accumulation of soluble sugars and amylopectin. For purple waxy maize, appropriately applying N also boosted the activities of enzymes involved in anthocyanin biosynthesis (PAL, F3H, and DFR), and due to providing a relatively greater amount of C backbones, significantly facilitated the accumulation of anthocyanins. Timely harvesting, while ensuring edibility, may contribute to maintaining the quality of purple waxy corn, providing consumers with more nutritional components and fewer harmful substances. This study provides clear scientific evidence for the rational N application and harvesting practices for waxy maize, particularly purple waxy corn, laying a solid foundation for sustainable agricultural development.

## Figures and Tables

**Table 1:** ANOVA results of investigating the traits of JN20 kernels at different filling stages under four N application rates.

Trait	*F* Values						
	Year (Y)	Nitrogen Level (NL)	Filling Stage (FS)	Y × NL	Y × FS	NL × FS	Y × NL × FS
Anthocyanin content (ANC)	2.92 *	3.85 **	4.16 **	0.01 ^ns^	0.08 ^ns^	0.16 ^ns^	0.07 ^ns^
Anthocyanin accumulation amount (AAA)	3.45 *	15.84 **	384.87 **	0.06 ^ns^	2.22 ^ns^	0.41 ^ns^	0.04 ^ns^
Total nitrogen content (TNC)	5.53 **	63.28 **	637.92 **	0.28 ^ns^	0.51 ^ns^	0.62 ^ns^	0.09 ^ns^
Nitrite content (NC)	0.20 ^ns^	104.91 **	1723.00 **	1.00 ^ns^	1.03 ^ns^	6.82 **	0.12 ^ns^
Lysine content (LC)	21.26 **	93.70 **	1365.30 **	1.45 ^ns^	0.05 ^ns^	2.00 ^ns^	1.93 ^ns^
Soluble sugar content (SSC)	0.05 ^ns^	13.54 **	620.57 **	0.13 ^ns^	0.29 ^ns^	2.16 *	0.03 ^ns^
Amylopectin content (AC)	4.34 **	19.95 **	876.85 **	0.02 ^ns^	0.08 ^ns^	2.19 *	0.05 ^ns^
Nitrate reductase activity (NRA)	21.80 **	102.58 **	1150.63 **	6.80 **	0.23 ^ns^	2.81 **	1.20 ^ns^
Glutamine synthetase activity (GSA)	0.08 ^ns^	5.84 **	661.20 **	0.02 ^ns^	1.90 ^ns^	0.50 ^ns^	0.04 ^ns^
AGPase activity (AGPA)	17.47 **	10.24 **	435.23 **	0.27 ^ns^	0.63 ^ns^	0.19 ^ns^	0.07 ^ns^
SSS activity (SSSA)	8.65 **	3.53 **	31.57 **	0.01 ^ns^	0.03 ^ns^	0.04 ^ns^	0.01 ^ns^
SBE activity (SBEA)	2.75 ^ns^	6.74 **	567.21 **	0.14 ^ns^	0.47 ^ns^	0.82 ^ns^	0.03 ^ns^
DBE activity (DBEA)	11.50 **	22.11 **	1623.66 **	0.39 ^ns^	2.15 ^ns^	0.46 ^ns^	0.34 ^ns^
PAL activity (PALA)	88.09 **	1.45 ^ns^	533.19 **	0.23 ^ns^	0.03 ^ns^	94.29 **	6.25 **
F3H activity (F3HA)	600.51 **	677.05 **	2.21 ^ns^	44.53 **	13.79 **	222.70 **	28.67 **
DFR activity (DFRA)	413.34 **	85.22 **	10.40 **	14.36 **	2.45 ^ns^	5.23 **	2.43 ^ns^
ANS activity (ANSA)	48.52 **	646.90 **	7.57 **	4.25*	0.91 ^ns^	1.43 ^ns^	4.57 **
UFGT activity (UFGTA)	35.55 **	188.76 **	2.27 ^ns^	1.84 ^ns^	0.01 ^ns^	1.17 ^ns^	1.15 ^ns^
C/N ratio	0.42 ^ns^	1.51 ^ns^	1386.47 **	0.07 ^ns^	0.39 ^ns^	0.48 ^ns^	0.19 ^ns^

Note: ^ns^ denotes no significant differences, and * and ** signify significant differences at *p* < 0.05, and *p* < 0.01 level, respectively. The abbreviations in the table correspond to those presented below. The activities of PAL, F3H, FDR, ANS, and UFGT were analyzed using ANOVA at 15 DAP and 30 DAP.

**Table 2:** Effects of N application doses on dynamic changes in the anthocyanin biosynthesis enzyme activities in the fresh grains of purple waxy maize at different days after pollination.

Year	Time (d)	Nitrogen Treatment	PAL Activity (mU g^−1^)	F3H Activity (U g^−1^)	DFR Activity (mU g^−1^)	ANS Activity (U g^−1^)	UFGT Activity (mU g^−1^)
2018	15	N0	22.52 ± 1.89 b	304.76 ± 14.11 c	469.42 ± 24.26	1626.49 ± 22.72 c	4.18 ± 0.16 b
		N1	25.34 ± 1.58 b	445.55 ± 7.11 a	581.52 ± 29.96	1558.18 ± 29.42 c	4.04 ± 0.13 b
		N2	33.17 ± 3.38 a	406.83 ± 11.71 b	411.13 ± 24.26	2083.46 ± 39.34 a	5.43 ± 0.23 a
		N3	31.39 ± 1.54 a	317.08 ± 10.01 c	434.45 ± 22.4	2003.37 ± 52.09 b	5.58 ± 0.28 a
		Average	28.10 ± 0.80	368.55 ± 3.3	474.13 ± 9.92	1817.88 ± 22.37	4.81 ± 0.16
			(2.86%)	(0.90%)	(2.09%)	(1.23%)	(3.28%)
	30	N0	50.59 ± 3.02 a	532.37 ± 10.31 a	607.52 ± 24.41	1419.21 ± 62.81 a	3.14 ± 0.22 b
		N1	46.52 ± 1.36 a	525.91 ± 8.07 a	616.49 ± 36.19	1447.48 ± 36.72 a	3.57 ± 0.08 a
		N2	40.47 ± 3.99 b	486.61 ± 5.66 b	494.53 ± 25.85	1237.84 ± 53.51 b	2.99 ± 0.19 b
		N3	38.59 ± 2.39 b	499.52 ± 9.31 b	490.05 ± 16.14	1134.19 ± 63.34 b	3.06 ± 0.18 b
		Average	44.05 ± 1.25	511.10 ± 0.67	552.15 ± 7.44	1309.68 ± 37.77	3.19 ± 0.06
			(2.83%)	(0.13%)	(1.35%)	(2.88%)	(1.89%)
2019	15	N0	26.8 ± 2.05 b	427.36 ± 12.32 c	599.45 ± 28.76 a	1723.07 ± 28.27 b	4.58 ± 0.29 b
		N1	29.51 ± 1.26 b	524.15 ± 12.32 a	624.56 ± 18.83 a	1798.45 ± 57.12 b	4.88 ± 0.10 b
		N2	40.16 ± 3.16 a	467.25 ± 5.66 b	580.62 ± 20.9 a	2236.57 ± 73.55 a	5.96 ± 0.27 a
		N3	42.14 ± 2.99 a	432.06 ± 5.66 c	615.59 ± 31.41 a	2147.06 ± 61.20 a	5.73 ± 0.12 a
		Average	34.65 ± 1.85	462.71 ± 2.83	605.06 ± 22.23	1976.29 ± 35.35	5.29 ± 0.05
			(5.35%)	(0.61%)	(3.67%)	(1.79%)	(0.91%)
	30	N0	58.32 ± 1.1 a	640.89 ± 5.38 a	740.24 ± 28.34 a	1534.63 ± 38.92 a	3.90 ± 0.11 ab
		N1	57.17 ± 1.75 a	549.38 ± 10.31 b	725.89 ± 24.41 a	1426.28 ± 60.38 b	4.20 ± 0.06 a
		N2	45.79 ± 2.09 b	507.14 ± 9.69 c	701.68 ± 24.41 a	1235.48 ± 74.78 c	3.22 ± 0.34 b
		N3	40.16 ± 2.08 c	512.42 ± 9.69 c	704.37 ± 14.82 a	1249.61 ± 44.13 c	3.31 ± 0.08 b
		Average	50.36 ± 0.41	552.46 ± 0.67	718.05 ± 9.25	1361.5 ± 17.7	3.66 ± 0.20
			(0.82%)	(0.12%)	(1.29%)	(1.30%)	(5.57%)

Note: The different lowercase letters denote significant differences (*p* < 0.05) between N treatments on the same day. The values in parentheses are the %CV of different N treatments on the same day.

## Data Availability

Dataset available upon request from the authors. The raw data supporting the conclusions of this article will be made available by the authors upon request.

## Supplementary Materials

The following supporting information can be downloaded at: https://www.mdpi.com/article/10.3390/plants13202882/s1, Figure S1: The overall changes of kernel anthocyanin accumulation amount (AAA) and content (ANC) under different N treatments and at different grain filling times in two years; Figure S2: The dynamic changes of anthocyanin accumulation rate in grains of purple waxy maize under different N treatments and at different grain filling times in two years; Figure S3: The overall changes of total nitrogen content (TNC) in grains of purple waxy maize under different N treatments and at different grain filling times in two years; Figure S4: The dynamic changes of N accumulation rate in grains of purple waxy maize under different N treatments and at different grain filling times in two years; Figure S5: The overall changes of nitrite content (NC) in grains of purple waxy maize under different N treatments and at different grain filling times in two years; Figure S6: The overall changes of lysine content (LC) in grains of purple waxy maize under different N treatments and at different grain filling times in two years; Figure S7: The dynamic changes of lysine accumulation rate in grains of purple waxy maize under different N treatments and at different grain filling times in two years; Figure S8: The overall changes of nitrate reductase (NR) and glutamine synthetase (GS) activities under different N treatments and at different grain filling times in two years; Figure S9: The overall changes of soluble sugar content (SSC) in grains of purple waxy maize under different N treatments and at different grain filling times in two years; Figure S10: The overall changes of amylopectin content (AC) in grains of purple waxy maize under different N treatments and at different grain filling times in two years; Figure S11: The dynamic changes of amylopectin accumulation rate in grains of purple waxy maize under different N treatments and at different grain filling times in two years; Figure S12: The overall changes of the enzymatic activity of carbon metabolism in fresh grains of purple waxy maize under different N treatments and at different grain filling times in two years; Figure S13: The overall changes of C/N ratio in grains of purple waxy maize under different N treatments and at different grain filling times in two years; Figure S14: Regression analysis of nitrogen, lysine, and soluble sugar content with anthocyanin content in grains of purple waxy maize under different N treatments and at different grain filling times in two years.
