# Supplementary material for: Nitrogen Level Impacts the Dynamic Changes in Nitrogen Metabolism, and Carbohydrate and Anthocyanin Biosynthesis Improves the Kernel Nutritional Quality of Purple Waxy Maize
Source: Plants (Basel). 2024 Oct 15;13(20):2882. doi: 10.3390/plants13202882 (PMC11510902; doi:10.3390/plants13202882)
Supplement: Supplementary file 1 [file plants-13-02882-s001.zip › plants-3202072-supplementary.pdf]

Supporting Information

Nitrogen Level Impacts the Dynamic Changes in Nitrogen Metabolism, and Carbohydrate and Anthocyanin Biosynthesis Improves the Kernel Nutritional Quality of Purple Waxy Maize

Wanjun Feng [1,†], Weiwei Xue [2,†], Zequn Zhao [2,†], Haoxue Wang [2], Zhaokang Shi [2], Weijie Wang [1,2], Baoguo Chen [2], Peng Qiu [1], Jianfu Xue [2,*] and Min Sun [2,*]

1 Sorghum Research Institute, Shanxi Agricultural University, Jinzhong 030600, China; fengwj123@sxau.edu.cn (W.F.); wangweijie0628@163.com (W.W.); qiupeng072954@163.com (P.Q.)

2 College of Agriculture, Shanxi Agricultural University, Jinzhong 030801, China; z20223184@stu.sxau.edu.cn (W.X.); zhaozequnsd@163.com (Z.Z.); z20223217@stu.sxau.edu.cn (H.W.); shishizhaokang@163.com (Z.S.); bgchen108@sina.com (B.C.)

* Correspondence: fudange95@163.com (J.X.); sm_sunmin@126.com (M.S.)

† These authors contributed equally to this work.

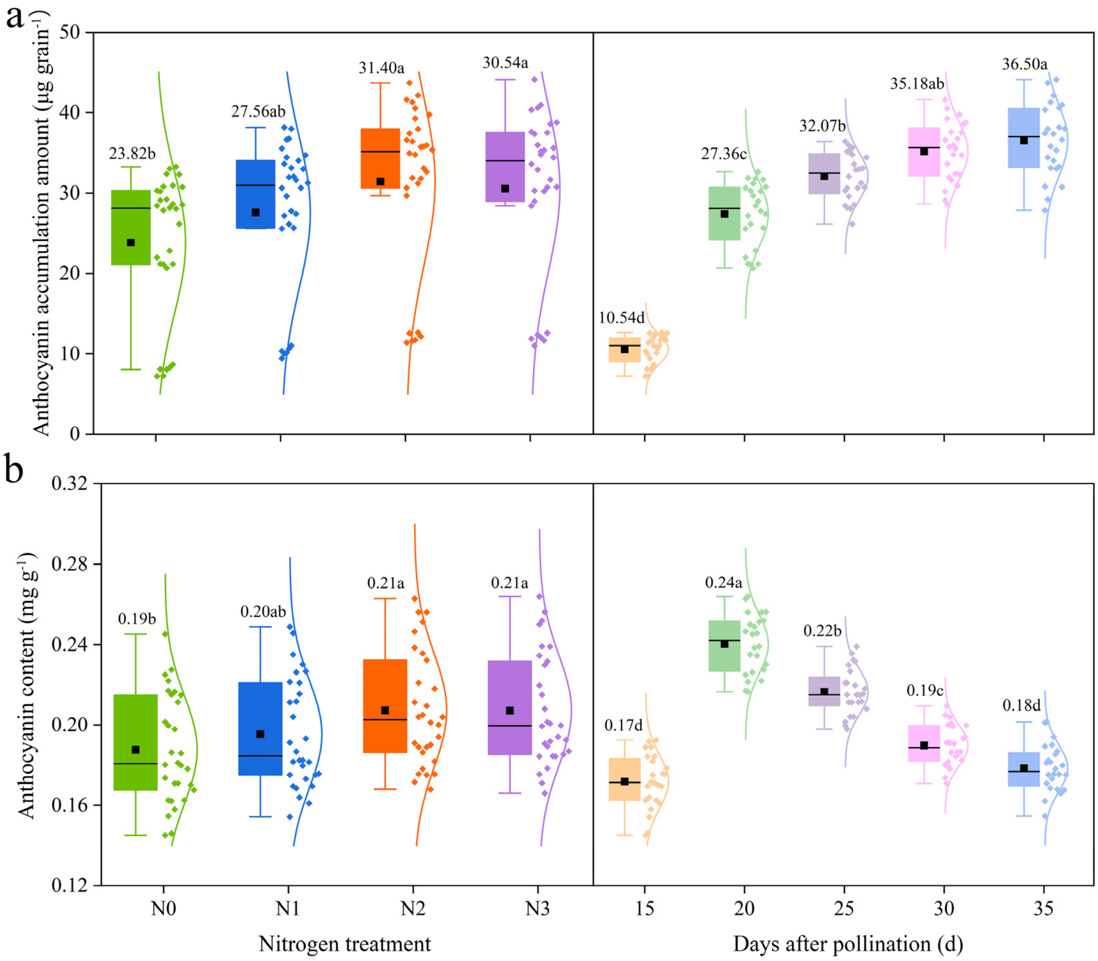

Figure S1. The overall changes of kernel anthocyanin accumulation amount (AAA) and content (ANC) under different N treatments and at different grain filling times in

two years. a, anthocyanin accumulation amount during 2018–19; b, anthocyanin content during 2018–19. The different lowercase letters denote significant differences at $p < 0.05$ level. The error bar denotes standard error based on 3 data.

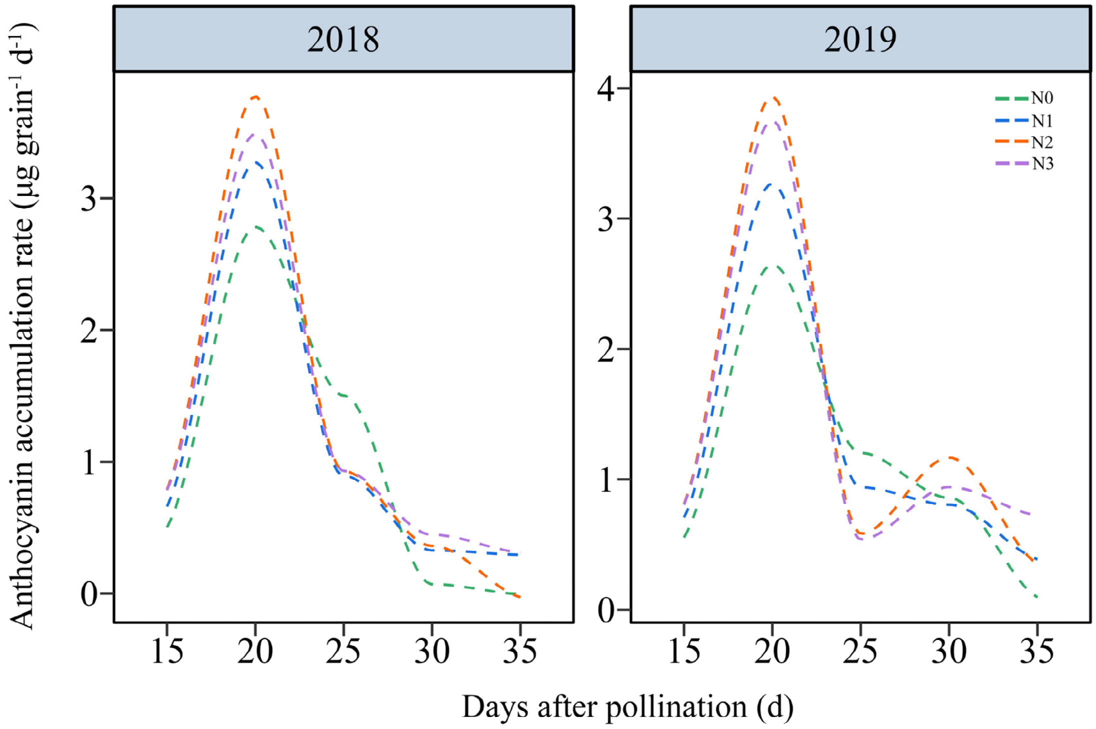

Figure S2. The dynamic changes of anthocyanin accumulation rate in grains of purple waxy maize under different N treatments and at different grain filling times in two years. The anthocyanin accumulation rate is the derivative of the grain accumulation content accumulation.

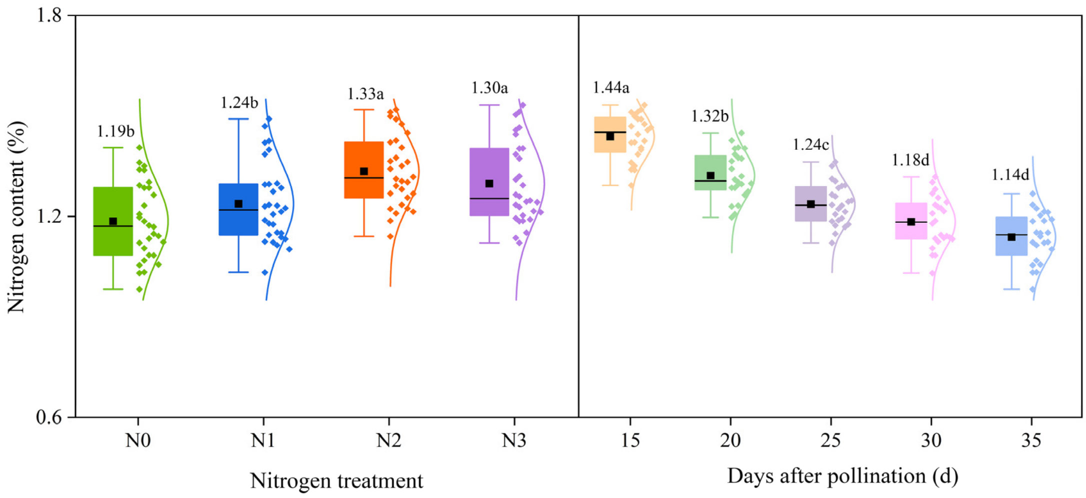

Figure S3. The overall changes of total nitrogen content (TNC) in grains of purple waxy maize under different N treatments and at different grain filling times in two years. The different lowercase letters denote significant differences at $p < 0.05$ level. The error bar denotes standard error based on 3 data.

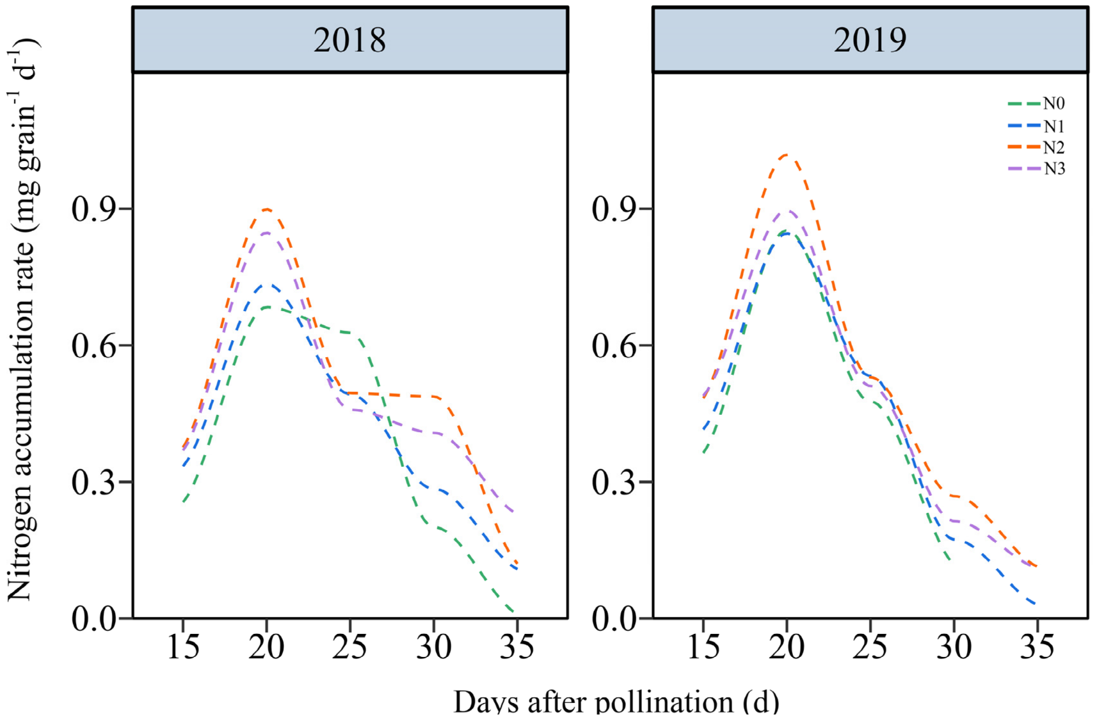

Figure S4. The dynamic changes of N accumulation rate in grains of purple waxy maize under different N treatments and at different grain filling times in two years. The N accumulation rate is the derivative of the grain N content accumulation.

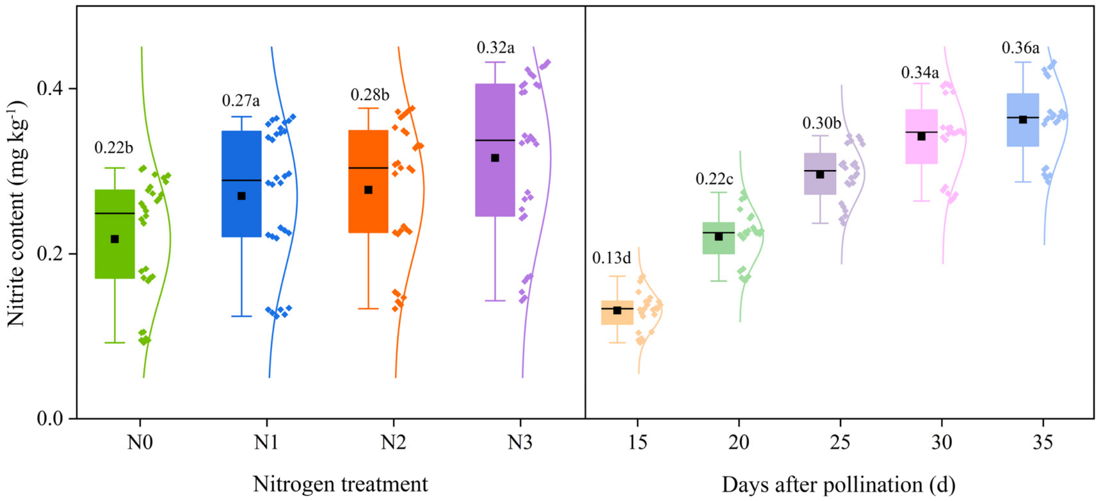

Figure S5. The overall changes of nitrite content (NC) in grains of purple waxy maize under different N treatments and at different grain filling times in two years. The different lowercase letters denote significant differences at $p < 0.05$ level. The error bar denotes standard error based on 3 data.

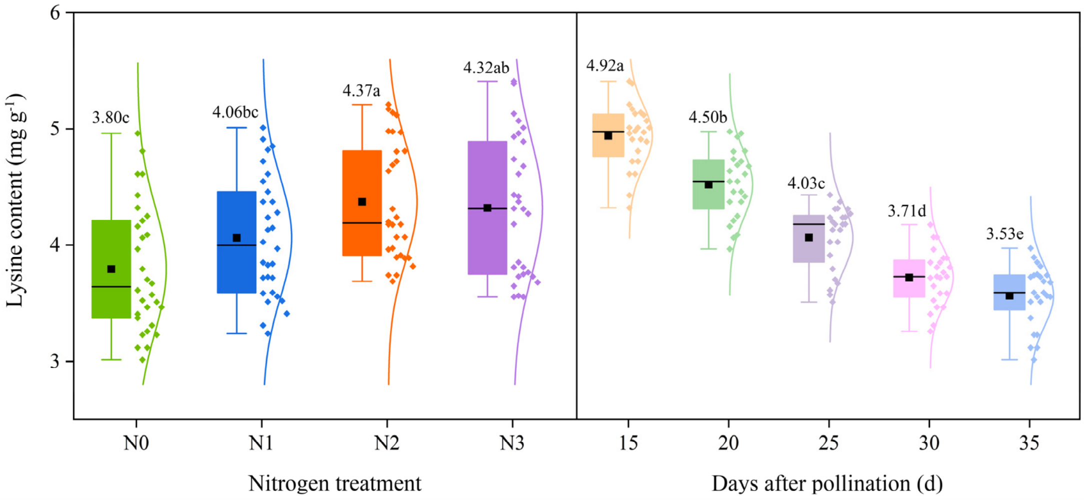

Figure S6. The overall changes of lysine content (LC) in grains of purple waxy maize under different N treatments and at different grain filling times in two years. The different lowercase letters denote significant differences at $p < 0.05$ level. The error bar denotes standard error based on 3 data.

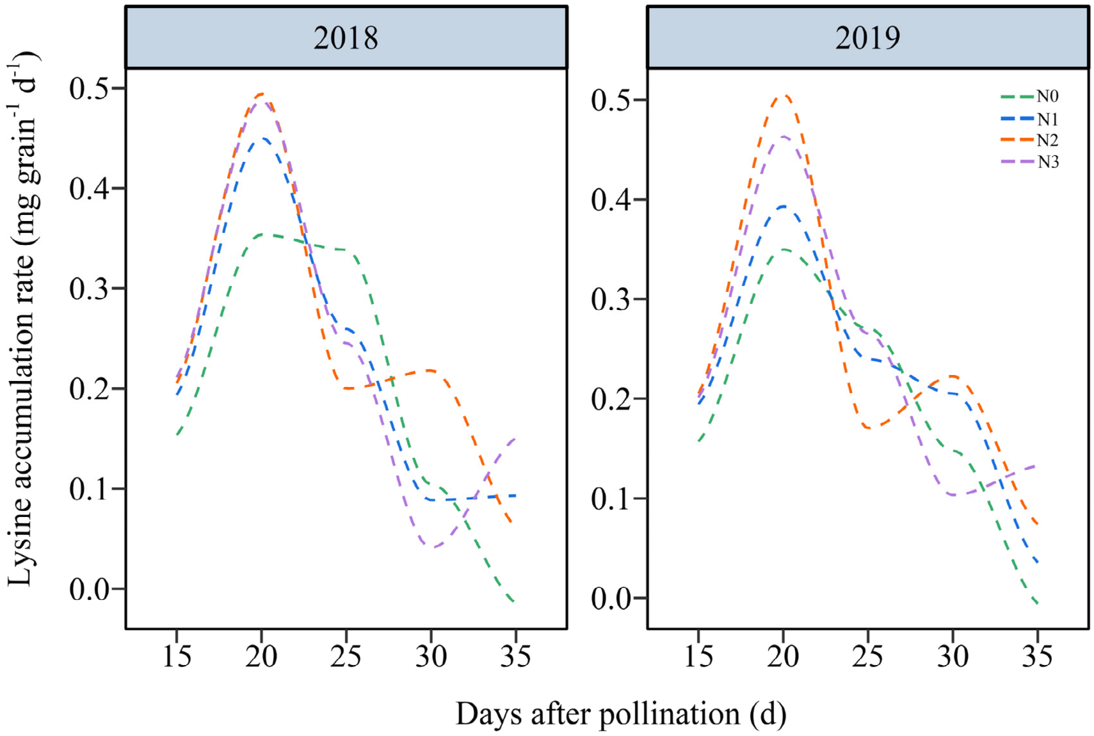

Figure S7. The dynamic changes of lysine accumulation rate in grains of purple waxy maize under different N treatments and at different grain filling times in two years. The lysine accumulation rate is the derivative of the grain lysine content accumulation.

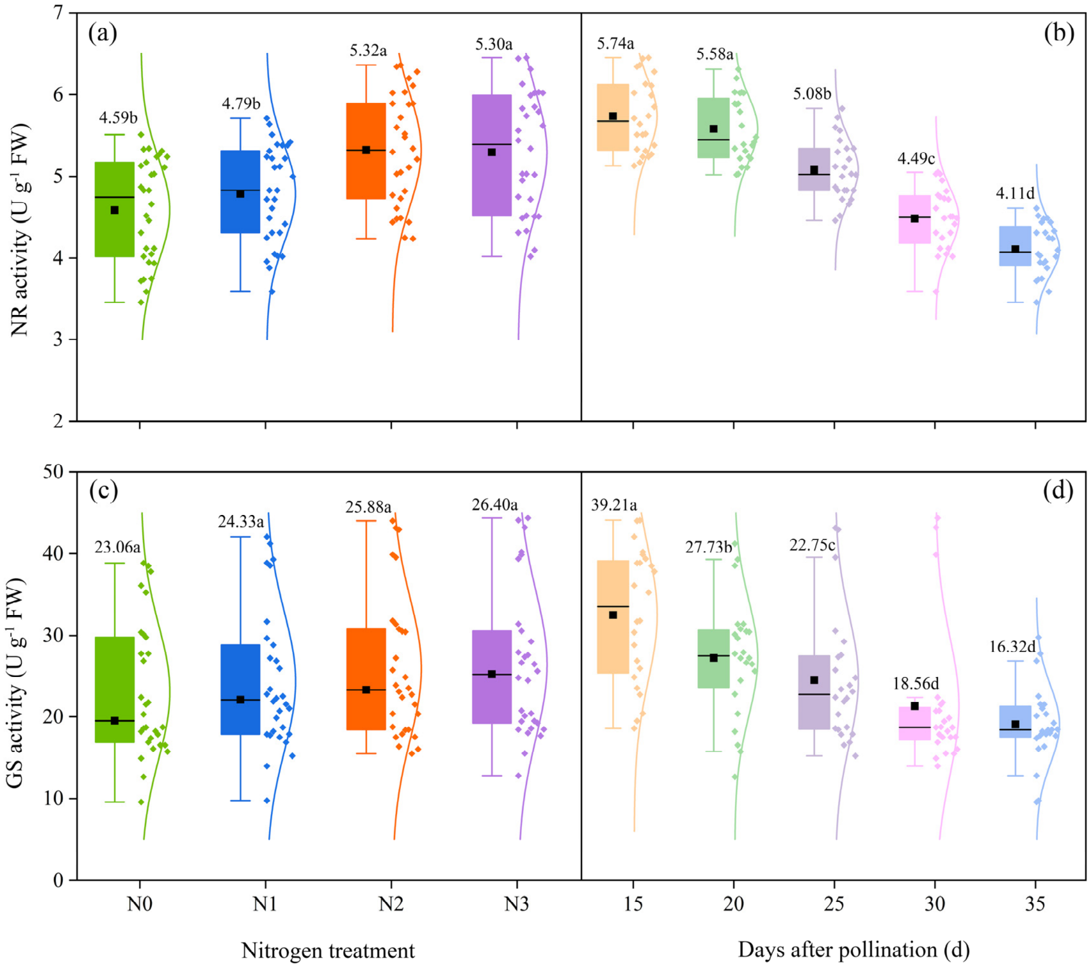

Figure S8. The overall changes of nitrate reductase (NR) and glutamine synthetase (GS) activities under different N treatments and at different grain filling times in two years. a and b, NR activity during 2018–19; c and d, GS activity during 2018–19. The different lowercase letters denote significant differences at $p < 0.05$ level. The error bar denotes standard error based on 3 data.

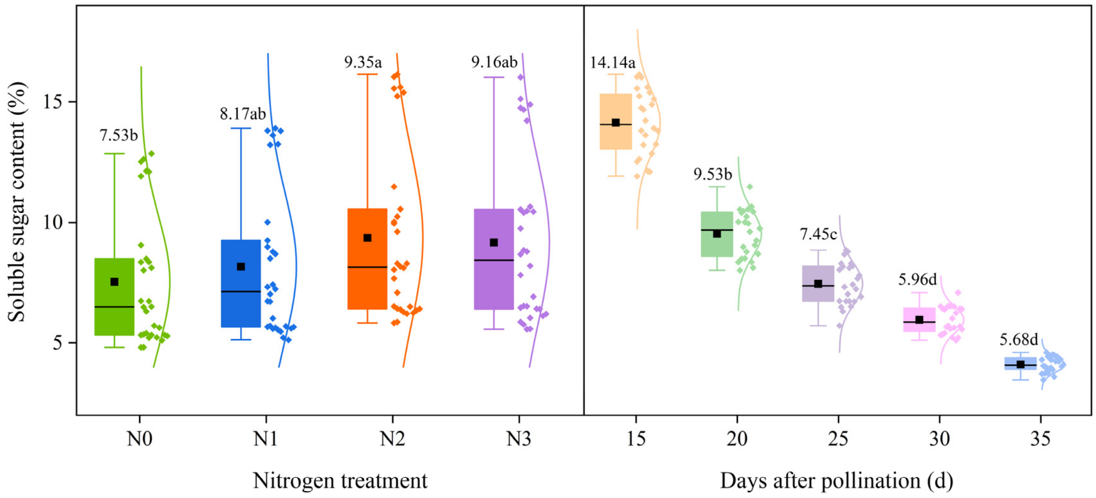

Figure S9. The overall changes of soluble sugar content (SSC) in grains of purple waxy maize under different N treatments and at different grain filling times in two years. The different lowercase letters denote significant differences at $p < 0.05$ level. The error bar denotes standard error based on 3 data.

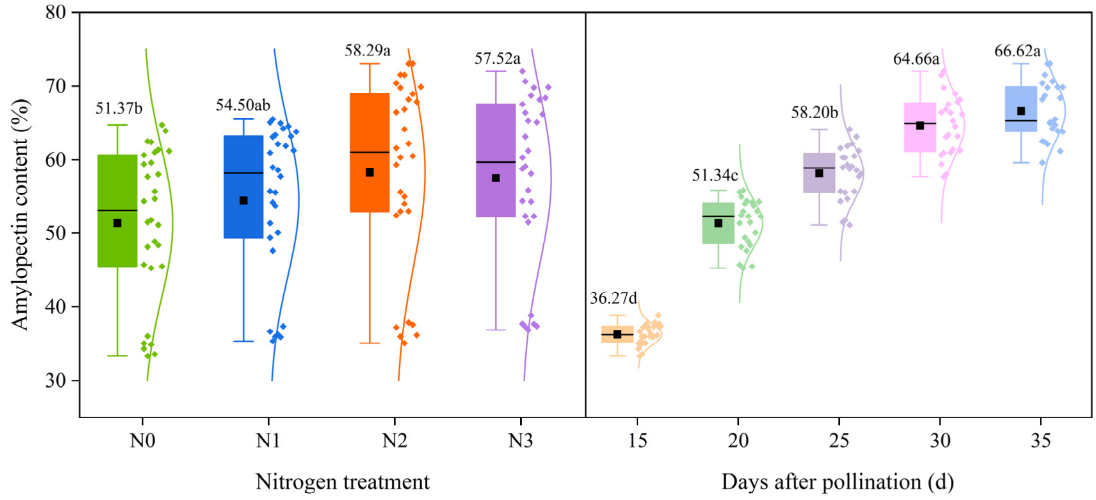

Figure S10. The overall changes of amylopectin content (AC) in grains of purple waxy maize under different N treatments and at different grain filling times in two years. The different lowercase letters denote significant differences at $p < 0.05$ level. The error bar denotes standard error based on 3 data.

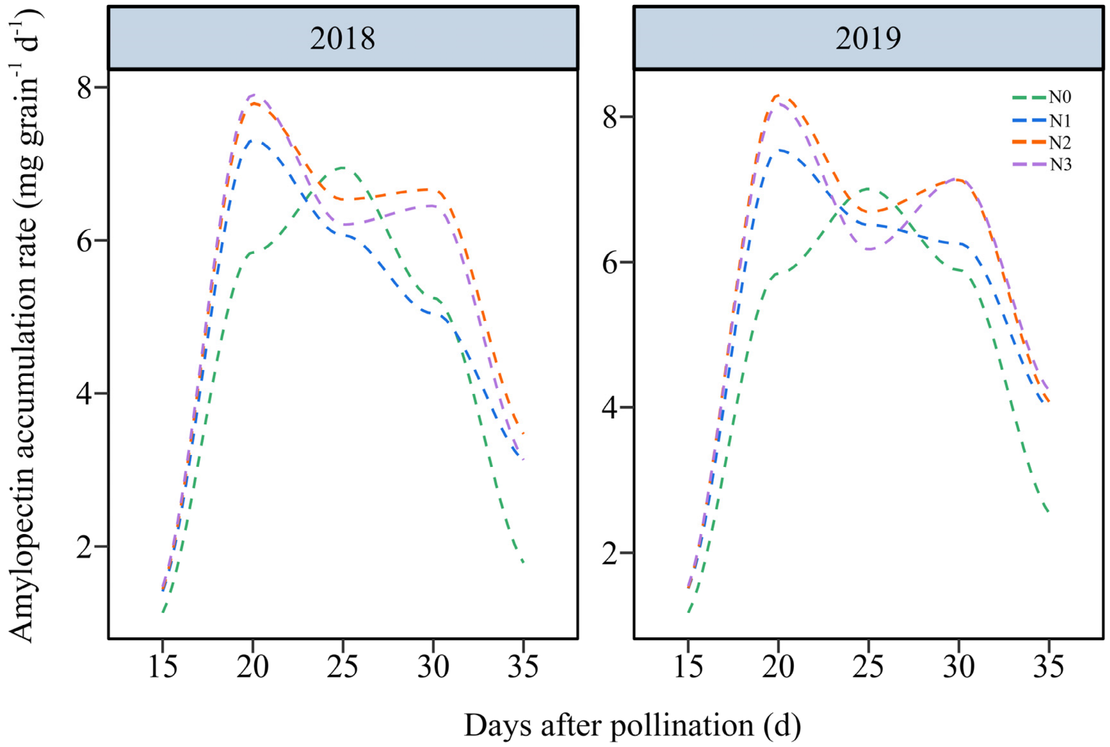

Figure S11. The dynamic changes of amylopectin accumulation rate in grains of purple waxy maize under different N treatments and at different grain filling times in two years. The amylopectin accumulation rate is the derivative of the grain amylopectin content accumulation.

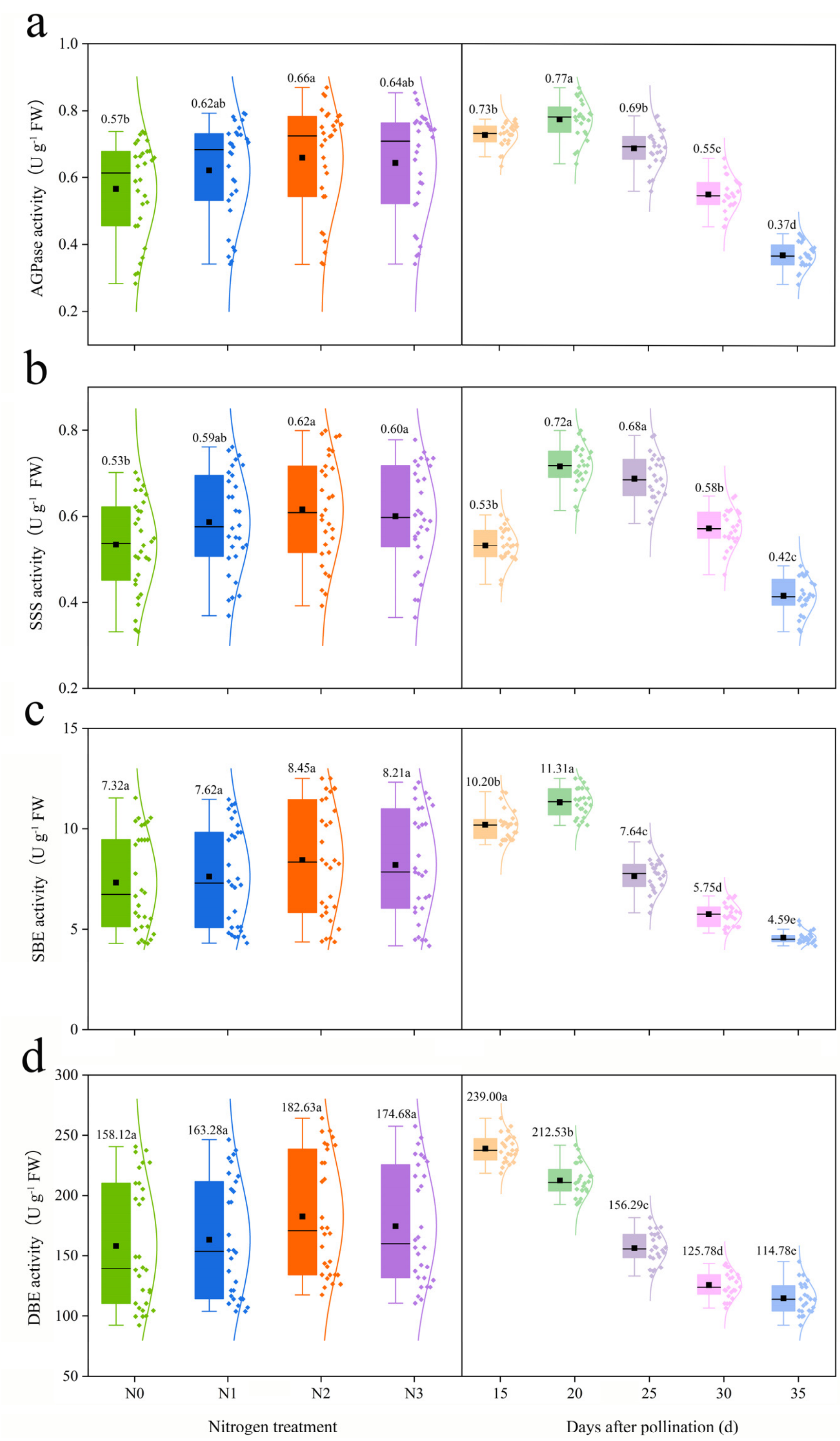

Figure S12. The overall changes of the enzymatic activity of carbon metabolism in fresh grains of purple waxy maize under different N treatments and at different grain filling times in two years. The different lowercase letters denote significant differences at $p < 0.05$ level. The error bar denotes standard error based on 3 data. a-d: ADP-glucose pyrophosphorylase (AGPase) activity, soluble starch synthase (SSS) activity, starch branching enzyme (SBE) activity and starch-debranching enzyme (DBE) activity of purple waxy maize at different days after pollination in 2018 and 2019, respectively.

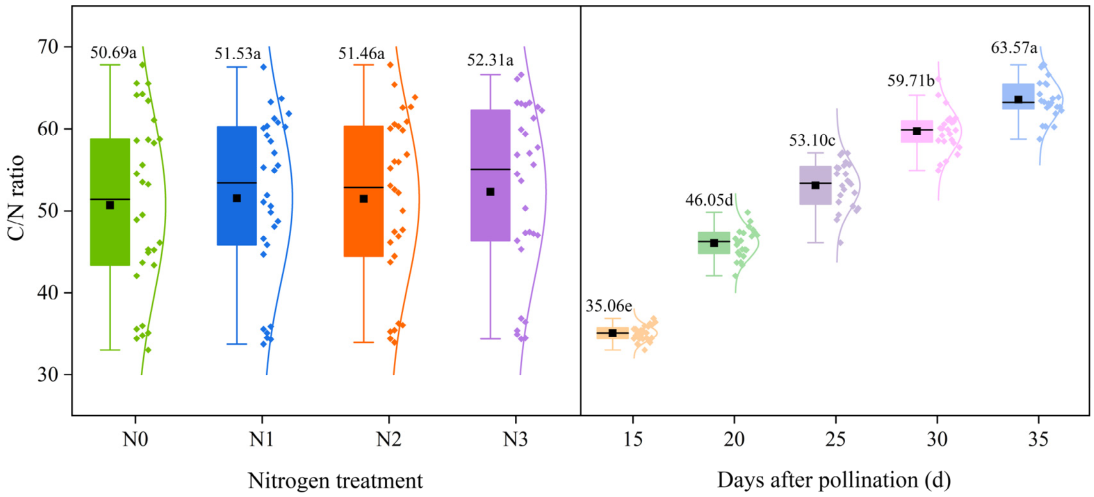

Figure S13. The overall changes of C/N ratio in grains of purple waxy maize under different N treatments and at different grain filling times in two years. The different lowercase letters denote significant differences at $p < 0.05$ level. The error bar denotes standard error based on 3 data.

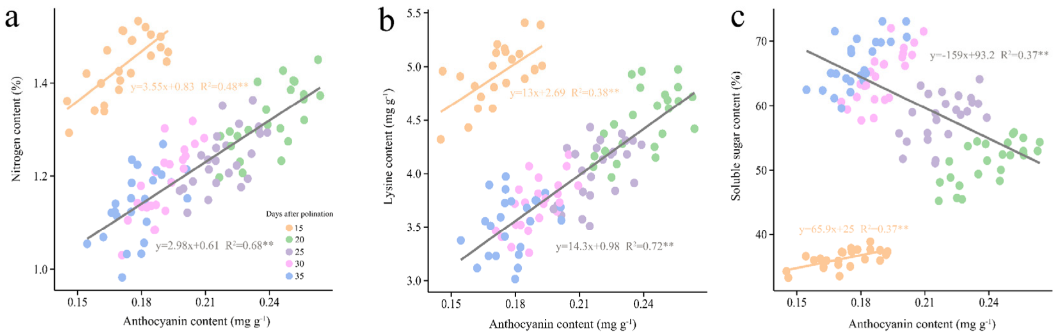

Figure S14. Regression analysis of nitrogen, lysine, and soluble sugar content with anthocyanin content in grains of purple waxy maize under different N treatments and at different grain filling times in two years. ** denotes significant differences at $p < 0.01$ level. The yellow regression line represents data from 15 days after pollination, while the dark gray regression line represents data from 20 to 35 days after pollination.
